# Host–Pathogen Dual Targeting With Repurposed Drugs Identifies a Synergistic Therapy for Intracellular *Staphylococcus aureus*

**DOI:** 10.1002/mbo3.70317

**Published:** 2026-05-28

**Authors:** Blanca Lorente‐Torres, Helena Á. Ferrero, Pablo Castañera, Jesús Llano‐Verdeja, Sergio Fernández‐Martínez, Amanda Herrero‐González, Farzaneh Javadimarand, Roberto López, Jesús F. Aparicio, Andrew M. Edwards, Volker Behrends, Luis M. Mateos, Álvaro Mourenza, Michal Letek

**Affiliations:** ^1^ Departamento de Biología Molecular, Área de Microbiología Universidad de León León Spain; ^2^ Departamento de Biología Molecular, Área de Biología Celular Universidad de León León Spain; ^3^ Departamento de Química y Física Aplicadas, Área de Química Física Universidad de León León Spain; ^4^ Centre for Bacterial Resistance Biology Imperial College London London UK; ^5^ Department of Infectious Disease Imperial College London London UK; ^6^ School of Medicine and Biosciences University of West London London UK; ^7^ Instituto de Biología Molecular, Genómica y Proteómica (INBIOMIC) Universidad de León León Spain; ^8^ Centro Interdisciplinar de Química e Bioloxía (CICA) Universidade da Coruña A Coruña Spain; ^9^ Grupo EXPRELA Instituto de Investigación Biomédica de A Coruña (INIBIC) A Coruña As Xubias Spain; ^10^ Instituto de Desarrollo Ganadero y Sanidad Animal (INDEGSAL) Universidad de León León Spain

**Keywords:** antimicrobial resistance, combination therapy, drug repurposing, intracellular infection, larvae, mice, *Staphylococcus aureus*

## Abstract

*Staphylococcus aureus* is a major cause of severe infections, including pneumonia and sepsis, partly due to its ability to survive within host cells where many antibiotics are ineffective. Drug repurposing offers a rapid strategy to identify compounds that enhance intracellular antibacterial activity by modulating host pathways. Here, a high‐throughput screen of 6297 clinically approved compounds in *S. aureus*‐infected A549 cells identified 5‐fluoro‐2′‐deoxycytidine (5‐FdC) as an effective intracellular inhibitor. When combined with rifapentine (5FR), 5‐FdC displayed synergistic activity across community‐ and hospital‐acquired MRSA and MSSA strains, as well as in different host cell types, including non‐tumorigenic bronchial cells. Metabolomic and host RNA‐sequencing analyses showed that 5‐FdC treatment activated host stress‐response and DNA damage response (DDR) pathways while restoring infection‐induced metabolic imbalances, particularly in amino acid and central carbon metabolism. These transcriptional and metabolic changes correlated with reduced intracellular bacterial markers. In vivo, the 5FR combination significantly decreased bacterial loads in *Galleria mellonella* and murine pneumonia models without detectable toxicity. This study presents the largest repurposing screen performed against intracellular *S. aureus* and identifies a synergistic host‐ and pathogen‐targeted combination that enhances bacterial clearance through coordinated modulation of host DDR, stress, and metabolic responses.

## Graphical abstract

A large intracellular drug‐repurposing screen identifies a potent synergy between 5‐fluoro‐2′‐deoxycytidine and rifapentine, acting through dual host–pathogen targeting to reduce *Staphylococcus aureus* burdens across strains, cell types, and infection models (created with BioRender).

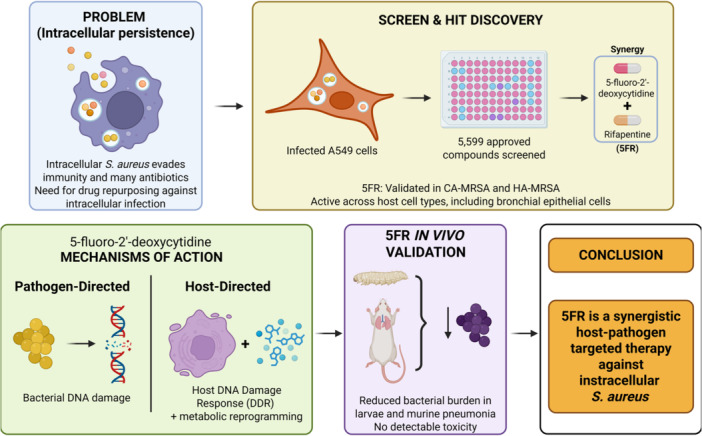

## 1 Introduction

*Staphylococcus aureus* is a facultative intracellular pathogen carried by ~30% of the human population (Tong et al. 2015; Tripathi 2025). It represents the leading bacterial cause of death across 135 countries and is a major etiological agent of pneumonia and bacteremia, accounting for more than one million deaths annually worldwide (Ikuta et al. 2022; Murray et al. 2022; Naghavi et al. 2024). Nasal colonization, facilitated by epithelial cell internalization, increases the risk of subsequent bacteremia in carriers (McEwen and Collignon 2018; Bravo‐Santano, Behrends, et al. 2019). Its intracellular lifestyle has also been linked to endocarditis, osteomyelitis, and even certain cancers (Hajam and Liu 2024; Lorente‐Torres et al. 2024; Odunitan et al. 2024; Volk et al. 2024).

Once internalized, *S. aureus* is effectively sheltered from host immune surveillance and from many antibiotics that were originally developed and optimized to act in extracellular environments (Volk et al. 2024). The bacterium can escape the phagosome and persist in the cytoplasm or within autophagosomes, where antibiotic access is severely restricted (Bravo‐Santano et al. 2018). This intracellular refuge, combined with a sophisticated arsenal of immune‐modulating virulence factors, enables long‐term persistence and relapse despite antibiotic regimens that should, in principle, be effective (Hajam and Liu 2024).

Although numerous antibiotics are available, their clinical impact is limited by two compounding problems. First, the misuse and overuse of antimicrobials have driven the rise of multidrug‐resistant (MDR) *S. aureus* strains (Lorente‐Torres et al. 2024). Second, and often underappreciated, many approved antibiotics show inadequate penetration into the intracellular compartments where the pathogen resides (Lehar et al. 2015). As a result, even antibiotics to which *S. aureus* is nominally susceptible may fail to reach bactericidal concentrations at the actual site of infection. Vaccines have also failed to provide protection against *S. aureus*, further underscoring the therapeutic gap (Hajam and Liu 2024). Meanwhile, the development of new antibiotics continues to decline because short‐course antimicrobials offer limited financial incentives compared with chronic therapies (Plackett 2020).

Given the shrinking effectiveness of conventional approaches, alternative strategies are urgently needed. Drug repurposing, *i.e*. re‐evaluating approved drugs for new anti‐staphylococcal applications, has emerged as a pragmatic and accelerated route to expand the therapeutic landscape (Das et al. 2016; Sun et al. 2016; Konreddy et al. 2018; Pushpakom et al. 2018; Schor and Einav 2018; Zheng et al. 2018; Miró‐Canturri et al. 2019; Lorente‐Torres et al. 2024). Repurposed compounds benefit from established safety, pharmacokinetics, and manufacturing pipelines, substantially reducing the time and cost associated with early‐stage drug development (Konreddy et al. 2018; Tiberi et al. 2018; Quezada et al. 2020). Importantly, repurposing can reveal novel mechanisms of action, providing opportunities to circumvent resistance and target the intracellular niche more effectively (Jampilek 2022).

Drug repositioning has already shown considerable promise against *S. aureus*, with multiple classes of approved agents displaying antimicrobial or antibiofilm activity. Notable examples include antipsychotics such as chlorpromazine and thioridazine (Martins et al. 2004; Czyż et al. 2014; Lorente‐Torres et al. 2024), selamectin (Folliero et al. 2023), NSAIDs like celecoxib and diflunisal, and redox‐active compounds such as ebselen (Thangamani et al. 2015a, 2015b; Hendrix et al. 2016). Other repurposed candidates include floxuridine and streptozotocin, which inhibit the *S. aureus* SaeRS two‐component system (Yeo et al. 2018), ibuprofen as a biofilm disruptor (Oliveira et al. 2019), sorafenib derivatives with anti‐MRSA and antibiofilm activity (Chang et al. 2016; Le et al. 2020), eltrombopag with in vivo anti‐biofilm efficacy (Lee et al. 2021; She et al. 2022), and anthelmintics such as niclosamide and oxyclozanide with potent anti‐MRSA activity (Rajamuthiah et al. 2015). More recently, rifabutin has been explored in MRSA osteomyelitis and periprosthetic joint infection models (Albano et al. 2019; Karau et al. 2020; Abad et al. 2021), while AZD‐5991 and TAK‐285 have been identified as novel inhibitors of *S. aureus* growth and biofilms (Huang et al. 2024; Tang et al. 2025).

Together, these studies highlight the therapeutic breadth that drug repurposing can offer, ranging from antibiofilm compounds to virulence modulators and membrane‐active drugs. However, despite this growing repertoire, almost all repurposed agents have only been tested in extracellular or biofilm assays. To date, only chlorpromazine and thioridazine have been evaluated in *bona fide* intracellular infection models of *S. aureus*, leaving the intracellular niche, a key reservoir in persistent and relapsing disease, largely unexplored.

Relying on repurposed drugs as monotherapies is also insufficient due to the persistent risk of resistance. A more effective route involves testing rational combinatorial therapies. Drug combinations can increase efficacy, lower required doses, and reduce toxicity (Duarte and Vale 2022; Kumar et al. 2023). Synergistic interactions are particularly valuable against MDR intracellular pathogens because they can delay resistance development and reduce mortality compared to monotherapy (Dhanda et al. 2023; Kumar et al. 2023). Combinations targeting both host and pathogen are especially promising: host‐directed therapies (HDTs) modulate cellular pathways exploited by intracellular bacteria and can complement direct‐acting antimicrobials (Zheng 2025). Because HDTs do not target the pathogen directly, they exert minimal selective pressure on bacterial populations, making resistance far less likely to emerge while still enhancing the effectiveness of conventional antibiotics.

In this study, we performed a high‐throughput repurposing screen of 6297 compounds in an intracellular infection model of *S. aureus*. From this screen, we identified a synergistic combination between the nucleoside analog 5‐fluoro‐2'‐deoxycytidine and the antibiotic rifapentine. This drug combination exhibits robust antibacterial activity across diverse *S. aureus* strains and mammalian cell types. To investigate the underlying mechanisms, we combined infection assays with transcriptomic and metabolomic profiling as well as the analysis of DNA repair mutants, revealing complementary host‐ and pathogen‐directed effects. In particular, our results suggest that 5‐FdC engages DNA‐damage response (DDR) pathways that interface with interferon signaling and metabolic reprogramming. Importantly, the combination retained efficacy in both cell‐based and animal infection models, providing proof‐of‐concept for rational combinatorial strategies to overcome the limitations of monotherapies against intracellular *S. aureus*.

## 2 Materials and Methods

### 2.1 Bacterial Strains, Cell Lines and Culture Conditions

*S. aureus* strains (Table 1) were cultured on Brain Heart Infusion (BHI) agar or broth (Condalab, Spain) and incubated at 37°C, with shaking at 200 rpm for liquid cultures. Routinely, bacterial cells were grown from preinocula in 50 mL of BHI broth with shaking at 200 rpm at 37°C until reaching an optical density at 600 nm (OD_600_) of 1, then they were centrifuged and washed twice with phosphate‐buffered saline (PBS; Gibco—Fisher‐Scientific, Spain). Afterwards, the bacterial cells were resuspended in 1.5 mL of PBS supplemented with 20% glycerol (Sigma–Aldrich, Spain). Aliquots of 100 μL were stored at −80°C until further use. Colony Forming Units (CFU) per mL of the preinoculum were calculated by serial dilution plating.

**Table 1:** *S. aureus* strains used in this study.

Strain name	Description	References
*S. aureus* USA300 LAC	USA300 lineage reference strain; clinical Community‐Associated Methicillin‐Resistant *S. aureus* (CA‐MRSA; ST8)	Diep et al. (2006)
*S. aureus* USA300‐GFP	*S. aureus* USA300 pCL55–Ptet–gfpmut2, expressing *gfp* under the tetracycline promoter; resistant to chloramphenicol	Reichmann et al. (2014)
*S. aureus* NCTC 13626	NCTC reference strain; MRSA TW20 (ST239‐III), healthcare‐associated Methicillin‐Resistant *S. aureus* (HA‐MRSA)	Holden et al. (2010)
*S. aureus* ATCC 25923	ATCC reference strain; quality‐control strain for antibiotic susceptibility testing (Methicillin‐Susceptible *S. aureus*, MSSA)	Treangen et al. (2014)
*S. aureus* NCTC 8325	Laboratory reference strain; parent of NCTC 8325‐4 and RN4220	Herbert et al. (2010)
*S. aureus* RN4220	NCTC 8325‐4 derivative; restriction‐deficient cloning host; mutated in the type I restriction–modification gene *hsdR* (restriction subunit)	Nair et al. (2011)
*S. aureus* USA300 JE2	*S. aureus* USA300 LAC derivative; parental background for the Nebraska Transposon Mutant Library (NTML)	Fey et al. (2013)
*S. aureus* USA300 JE2 pCN34 P*recA‐gfp*	*S. aureus* USA300 JE2 containing pCN34 with *gfp* under the control of the *recA* promoter; resistant to kanamycin	Clarke et al. (2019)
*S. aureus* NE11	*S. aureus* USA300 JE2 *recJ::Tn* (NTML); transposon insertion in the *recJ* gene encoding the single‐stranded DNA‐specific exonuclease RecJ.	Fey et al. (2013)
*S. aureus* NE145	*S. aureus* USA300 JE2 *uvrA::Tn* (NTML); transposon insertion in the *uvrA* gene encoding the nucleotide excision repair protein UvrA.	Fey et al. (2013)
*S. aureus* NE445	*S. aureus* USA300 JE2 *umuC::Tn* (NTML); transposon insertion in the *umuC* gene encoding the error‐prone polymerase V subunit.	Fey et al. (2013)
*S. aureus* NE458	*S. aureus* USA300 JE2 *xseA::Tn* (NTML); transposon insertion in the *xseA* gene encoding the large subunit of exonuclease VII.	Fey et al. (2013)
*S. aureus* NE667	*S. aureus* USA300 JE2 *hsdR::Tn* (NTML); transposon insertion in the *hsdR* gene encoding the restriction subunit of the type I RM system.	Fey et al. (2013)
*S. aureus* NE883	*S. aureus* USA300 JE2 *xerC::Tn* (NTML); transposon insertion in the *xerC* gene encoding the site‐specific recombinase XerC.	Fey et al. (2013)
*S. aureus* NE949	*S. aureus* USA300 JE2 *hsdM::Tn* (NTML); transposon insertion in the *hsdM* gene encoding the modification subunit of the type I RM system.	Fey et al. (2013)
*S. aureus* NE982	*S. aureus* USA300 JE2 *hsdS::Tn* (NTML); transposon insertion in the *hsdS* gene encoding the specificity subunit of the type I RM system.	Fey et al. (2013)
*S. aureus* NE997	*S. aureus* USA300 JE2 *hemK (prmC)::Tn* (NTML); transposon insertion in the *hemK (prmC)* gene encoding a translation termination factor methyltransferase.	Fey et al. (2013)
*S. aureus* NE1012	*S. aureus* USA300 JE2 *rexB::Tn* (NTML); transposon insertion in the *rexB* gene encoding the exonuclease/helicase subunit of the RexAB complex.	Fey et al. (2013)
*S. aureus* NE1028	*S. aureus* USA300 JE2 *nfo::Tn* (NTML); transposon insertion in the *nfo* gene encoding the base‐excision repair endonuclease IV.	Fey et al. (2013)
*S. aureus* NE1212	*S. aureus* USA300 JE2 *uvrC::Tn* (NTML); transposon insertion in the *uvrC* gene encoding the nucleotide excision repair endonuclease UvrC.	Fey et al. (2013)
*S. aureus* NE1451	*S. aureus* USA300 JE2 *sbcC::Tn* (NTML); transposon insertion in the *sbcC* gene encoding the SbcC ATPase involved in DNA repair.	Fey et al. (2013)
*S. aureus* NE1824	*S. aureus* USA300 JE2 NTML mutant with a transposon insertion in an uncharacterized gene encoding a hypothetical protein.	Fey et al. (2013)
*S. aureus* NE1866	*S. aureus* USA300 JE2 *dinB::Tn* (NTML); transposon insertion in the *dinB* gene encoding the error‐prone DNA polymerase IV.	Fey et al. (2013)

For the in vitro infection assays, we used lung epithelial cells A549 (American Type Culture Collection [ATCC, USA], Ref. CCL‐185), immortalized primary bronchial epithelial cells modified with hTERT‐BMI (BEC, Applied Biological Materials, Spain, Ref. T0498), colon epithelial cells HCT116 (ATCC, USA, Ref. CCL‐247) and epithelial cancer cells derived from breast adenocarcinoma MCF7 (ATCC, USA, Ref. HTB‐22), all of them were cultured in 100 mm cell culture plates with Dulbecco's Modified Eagle's Medium (DMEM; Gibco, Thermo Fisher Scientific, USA) supplemented with pyruvate, glucose, glutamine, 10% heat‐inactivated fetal bovine serum (FBS; Gibco), and 5% penicillin‐streptomycin solution (Corning, USA). BECs were cultured on gelatin‐coated plates. The cell lines expressing constitutively mCherry were generated by transduction with the pCDH‐CMV‐mCherry‐T2A‐Puro vector (Addgene Ref. 72264) as previously described (Lorente‐Torres et al. 2025). To ensure selective growth of mCherry‐expressing cells, 1 μg/mL puromycin (Sigma–Aldrich, Spain) was added to the culture media. Cells were grown at 37°C in a 5% CO_2_ atmosphere. The expression of mCherry was confirmed by measuring fluorescence in a VICTOR Nivo Multimode Microplate Reader (Revvity, Spain) with an excitation filter of 580 nm and an emission filter of 625 nm (Lorente‐Torres et al. 2025).

### 2.2 High‐Throughput Screening

For the screening assay, two libraries of 6995 drugs in total (MedChemExpress, USA, Refs. HY‐L035P‐PartA and HY‐L021P‐PartA) were tested on A549 cells. Among these, 1396 entries (19.96%), corresponding to 698 compounds tested in duplicate, were identified by name and molecular formula. Of the 698 duplicated compounds, 94.84% displayed consistent biological effects, whereas 5.16% showed variability. After removing duplicates, a total of 6297 unique compounds were retained for further analysis.

Target analysis revealed that the majority of drugs were related to cancer (29.05%; Figure S1A). Mechanisms related to infections, including bacterial (7.73%), viral (3.67%), parasitic (1.29%), and fungal targets (1.2%; Figure S1B), accounted for 13.88% of the total. These were followed by targets associated with metabolic disease (13.47%), inflammation/immunology (12.5%), or neurological disease (12.31%) (Figure S1A).

Prior to infection, cells were seeded in black‐walled, flat‐bottomed 96‐well plates with tissue culture‐treated surfaces using DMEM supplemented with 10% FBS, but not with penicillin and streptomycin, at a cellular density of 8 × 10^4^ cells/mL and incubated for 24 h at 37°C with 5% CO_2_. *S. aureus* USA300 LAC aliquots were washed twice with PBS and adjusted to a final Multiplicity of Infection (MOI) of 1 and added to the cells. The plate was centrifuged at 800 × *g* for 5 min at room temperature and incubated at 37°C with 5% CO_2_ for 1 h. After the infection, compounds from the library were added at a final concentration of 10 µM along with gentamicin sulfate (100 µg/mL; MP Biomedicals, USA) to aid in the selection of intracellular bacteria. The plates were then incubated for 20 h at 37°C with 5% CO_2_. Plates were washed twice with PBS and read at 580/625 nm for quantification of mCherry fluorescence.

### 2.3 Minimum Inhibitory Concentrations and Synergy Testing

Minimum Inhibitory Concentrations (MIC) were tested following broth microdilution as previously described (Wiegand et al. 2008). Two‐fold serial dilutions of the selected compounds were prepared in Mueller‐Hinton Broth (MHB) in flat‐bottomed 96‐well plates. Wells were inoculated with *S. aureus* bacterial strains in the stationary phase at a final concentration of 1 × 10^5^ CFU/mL. Plates were incubated overnight at 37°C under static conditions. MIC was defined as the lowest compound concentration that prevented bacterial growth, as determined by measuring absorbance at 600 nm.

For synergy detection among our selected compounds, we faced two‐fold dilutions of each compound previously prepared separately in 96‐well plates, using MHB, following the checkerboard method (Hsieh et al. 1993). Similarly to MIC assays, wells were inoculated with a final concentration of 1 × 10^5^ CFU/mL from the stationary phase of *S. aureus* bacterial strains, incubated overnight at 37°C under static conditions. The following day, the MIC was defined as the lowest concentration as determined by measuring absorbance at 600 nm. Synergy was assessed using the checkerboard method (Hsieh et al. 1993), with the Fractional Inhibitory Concentration Index (FICI) calculated as: $\mathrm{FICI}=\left(\frac{\mathrm{MIC A combined}}{\mathrm{MIC A}}\right)+\left(\frac{\mathrm{MIC B combined}}{\mathrm{MIC B}}\right)$

Compound interactions were defined as follows: synergy was defined as FICI ≤ 0.5, while an additive effect was defined as FICI values comprised between 0.51 and 0.99, an indifferent effect as FICI values comprised between 1 and 2, and antagonism was defined as FICI ≥ 2 (Hsieh et al. 1993).

### 2.4 Antimicrobial Killing Assays

We performed bacterial killing assays as previously described (Sabnis et al. 2021). From overnight cultures grown in MHB, 1 mL of *S. aureus* was pelleted at 16,000 × *g* (2 min) and washed twice with 1 mL of fresh media. Then, 300 μL of a washed culture adjusted to a density of 1 × 10^7^ CFU/mL were added to 2.7 mL of MHB media containing the desired compound concentrations (MIC 1×‐8×). The samples were incubated at 37°C with 200 rpm shaking. At different time points (T0, T2, T4, T6, and T24), cultures were serially diluted 10‐fold in 200 µL of sterile PBS and plated on MHA for CFU enumeration.

### 2.5 Infection Assays

Combinations were tested in different cell lines as follows. Cell lines were seeded in 96‐well plates at a density of 2 × 10^4^ cells per well and incubated at 37°C with 5% CO_2_. Cells were infected using *S. aureus* aliquots at an MOI of 10 and incubated at 37°C with 5% CO_2_ for 1 h. To assess potential differences in response across different cell lines, the selected synergistic concentration was tested over a range of concentrations, from 16 times the concentration to 1/16 of the concentration. Two‐fold serial dilutions were prepared in advance in a separate flat‐bottomed 96‐well plate, using DMEM supplemented with 10% FBS and not containing antibiotics, starting from 16 times the selected concentration of the combination. After the incubation period, dilutions were added to the infected cells and incubated for 20 h at 37°C with 5% CO_2_. mCherry fluorescence measurements were taken at 580/625 nm after washing the plate twice with PBS.

### 2.6 In Vivo Larvae Testing

Compounds were tested in vivo on *Galleria mellonella* larvae (Hofkens et al. 2024). We estimated that a dose of 5 mg/kg 5FR in humans corresponds to approximately 1 µg/larva in *G. mellonella*, which is equivalent to 4 mg/kg in mice (Piatek et al. 2021).

200–500 mg larvae were placed in 100 mm Petri dishes at 10 larvae per plate with cellulose filter paper. Larvae were kept at room temperature until the next day, when they were incubated at 37°C for 1 h before infection.

Larvae were infected with overnight cultures on BHI or frozen preinocula of *S. aureus* USA300 LAC. An aliquot of 1 mL of the culture or the frozen preinocula was washed twice with PBS and adjusted to a final concentration of 1 × 10^5^ CFU/mL. Subsequently, 10 µL of the adjusted culture of *S. aureus* were injected with a U‐100 scale insulin syringe and 29 G Micro‐Fine needle (BD, USA) on the last right proleg of the larvae. Separately, a negative control group of infection received 10 µL of PBS in the first left proleg, while another negative control group remained unmanipulated. Melanisation and movement were used as measures of health (Hesketh‐Best et al. 2021).

When testing drug effectiveness, the larvae were incubated at 37°C for 1.5 h after infection. Then, 10 µL of the different treatments at their desired concentrations were injected into the first left proleg. PBS negative controls included injections of 20 µL of PBS, 10 µL in the first left proleg and 10 µL in the last right proleg. The larvae were kept at 37°C for 5 days, and their survival was measured to evaluate the effect of the treatments.

### 2.7 Murine Infection Model

All procedures received approval from the Ethics Committee on Animal Experimentation of the University of León and the regional authority *Junta de Castilla y León* (approval code OEBA‐ULE‐001‐2025). Experimental protocols complied with the ARRIVE guidelines, the European Union Directive 2010/63/EU, and Spanish legislation on animal experimentation (RD 53/2013). Every effort was made to reduce the number of mice employed and to prevent unnecessary distress.

*S. aureus* USA300 LAC was grown in BHI broth for 24 h at 37°C. Cultures were centrifuged at 3500 *× g* for 15 min, washed three times with PBS (pH: 7.4), and resuspended to 2 × 10^8^ CFU/20 µL for intranasal infection.

Male and female BALB/c mice (6–8 weeks old) were obtained from Charles River Laboratories and housed under specific pathogen‐free conditions in ventilated type III cages with food and water provided *ad libitum*. Humane endpoints were strictly applied, including euthanasia in cases of > 20% body weight loss, severe dehydration with signs of distress (arched back, sunken eyes, piloerection), or persistent diarrhea more than 48 h. Animals were euthanized by cervical dislocation before the onset of severe disease signs. These criteria were met in only one male mouse from the PBS control group.

After a 5‐day acclimatization period, mice were anesthetized intraperitoneally with dexmedetomidine (0.3 mg/kg) and ketamine (80 mg/kg) before intranasal infection with *S. aureus* USA300 LAC. Each animal received 20 µL of bacterial suspension (2 × 10^8^ CFU) in one nostril (Kim et al. 2014). Twenty‐four hours post‐infection, food and water were withdrawn for 5 h before treatment. Mice were randomly allocated into four groups (*n* = 7 per sex per group) and assigned to two conditions: (i) males and females infected, treated with PBS (control); (ii) males and females infected, treated orally with 5‐fluoro‐2′‐deoxycytidine (5‐FdC, 4 mg/kg) plus rifapentine (4 mg/kg) in combination with tetrahydrouridine (THU, 100 mg/kg). THU was included to inhibit cytidine deaminase, thereby preventing the rapid degradation of 5‐FdC and increasing its stability and bioavailability in vivo (Newman et al. 2015). Treatments were administered via oral gavage once daily for three consecutive days.

At 96 h post‐infection, mice were euthanized by cervical dislocation, and lungs and spleens were aseptically collected and weighed. Importantly, this method of euthanasia does not alter the number of bacteria recovered from the organs (Turner et al. 2011). Bacterial burden was quantified by quantitative PCR (qPCR). Organs were homogenized in sterile PBS and stored at –80°C until processing. For DNA purification, 200 µL of homogenate was transferred into FastPrep tubes containing matrix A (MP Biomedicals, Spain). Samples were supplemented with lysostaphin (50 µg/mL; Sigma–Aldrich, Spain) and lysozyme (1 mg/mL; Sigma–Aldrich, Spain) and incubated for 30 min at 37°C. After two bead‐beating cycles (30 s at 6.5 m/s, with cooling on ice between runs), samples were centrifuged briefly and processed with the Qiagen DNeasy Blood & Tissue Kit according to the manufacturer's instructions (Qiagen, Spain). DNA was eluted in 50 µL of nuclease‐free water and quantified using a NanoDrop (Thermo Fisher Scientific, Spain).

Bacterial DNA levels were determined using TB Green Premix Ex Taq (Takara, Japan). Reactions were prepared in 50 µL volumes containing 25 µL TB Green master mix (2×), 1 µL ROX reference dye (50×), 0.4 µM of each primer, and 5 µL of template DNA at 20 ng/µL. Amplification was performed on a QuantStudio 5 Real‐Time PCR System (Applied Biosystems, USA) with the following cycling program: initial denaturation at 95°C for 30 s, followed by 40 cycles of 95°C for 5 s and 60°C for 30 s, and a final melting curve analysis.

For *S. aureus* detection, primers targeting the *nuc* gene (F: 5′‐GCGATTGATGGTGATACGGTT‐3′, R: 5′‐AGCCAAGCCTTGACGAACTAAAGC‐3′; 279 bp amplicon) were used. As internal control, murine *Gapdh* was amplified (F: 5′‐TGTGTCCGTCGTGGATCTGA‐3′, R: 5′‐TTGCTGTTGAAGTCGCAGGAG‐3′; 150 bp amplicon). Relative bacterial loads were calculated using the ΔCt method (Ct_nuc – Ct_GAPDH), expressed as 2^–ΔCt.

### 2.8 Metabolomics

Metabolic profiling was carried out as previously described (Bravo‐Santano et al. 2018; Bravo‐Santano, Ellis, et al. 2019). A549 cells were seeded in 6‐well plates in DMEM + 10% FBS without antibiotics at a density of 5 × 10^5^ cells per well. Three experimental groups were established: (i) uninfected controls; (ii) cells infected with log‐phase *S. aureus* at an MOI of 10 and incubated at 37°C in 5% CO_2_ for 1 h, then exposed to gentamicin sulphate (100 µg/mL; MP Biomedicals, USA); and (iii) infected cells exposed to 5‐FdC (10 µM) in the presence of gentamicin sulphate (100 µg/mL). Following treatment, cells were further incubated for 24 h at 37°C in 5% CO_2_. After this, cells were then washed with 1 mL of ice‐cold Ringer's solution and quenched by adding 1 mL of cold (−20°C) methanol, detached by using a cell scraper and immediately stored at −80°C (Bravo‐Santano et al. 2018; Bravo‐Santano, Ellis, et al. 2019).

To obtain separated organic and aqueous fractions, we proceeded to a dual‐phase extraction, performed by adding 300 µL of CHCl_3_/MeOH (2:1) and vortexing for 30 s. After the addition of 300 µL of water and centrifugation (16,000 *× g*, 10 min, room temperature), the top aqueous layer was transferred into a glass vial and dried before being stored at −80°C. The lower organic layers were placed into glass vials and dried overnight before being stored at −80°C (Bravo‐Santano et al. 2018; Bravo‐Santano, Ellis, et al. 2019).

Aqueous fractions were derivatised in a two‐step process. First, fractions were resuspended in 20 µL of 20 mg/mL methoxyamine hydrochloride in anhydrous pyridine and kept at 30°C for 90 min, followed by the addition of 80 µL *N‐tert‐*Butyldimethylsilyl‐N‐methyltrifluoroacetamide (MTBSTFA) and derivatization at 70°C for 1 h (Kind et al. 2009).

From the organic fraction, both lipid‐bound and free fatty acids were analyzed. For the transmethylation of the lipid‐bound fatty acids, the dried fraction was reconstituted in a 300 µL methanol/toluene solution (1:1 ratio), treated with 200 µL of 0.5 M sodium methoxide, and incubated for 1 h at room temperature. The reaction was stopped by adding 500 µL of 1 M NaCl and 25 µL of concentrated HCl. The resulting samples were extracted using 500 µL of hexane, and the organic layers were dried in a fume cupboard under N_2_. Free fatty acids were then derivatized using 40 µL acetonitrile and 40 µL of MTBSTFA and incubated at 70°C for 1 h. Samples were finally centrifuged at 400 *× g* for 5 min before being transferred into a clean vial insert for Gas Chromatography‐Mass Spectrometry (GC‐MS) analysis (Bravo‐Santano et al. 2018; Bravo‐Santano, Ellis, et al. 2019).

GC‐MS analysis was performed on an Agilent 7890 gas chromatograph (Agilent Technologies, USA) equipped with a 30 m DB‐5MS capillary column with a 10 m Duraguard column connected to an Agilent 5975 MSD operating under electron impact ionization (Agilent Technologies, USA). Samples were injected into deactivated splitless liners using helium as the carrier gas (Kind et al. 2009). Metabolites were identified based on internal databases and quantified using an established workflow (Charidemou et al. 2024). Briefly, samples were deconvoluted in AMDIS (NIST, USA) (Stein 1999) and quantified using an in‐house script (Behrends et al. 2011).

### 2.9 RNA‐Sequencing

Total RNA from infected A549 cells, treated or untreated, was isolated with the RNeasy Mini Kit (Qiagen, Germany) following the manufacturer's instructions. RNA concentration and purity were determined using a NanoDrop (Thermo Fisher Scientific, USA) spectrophotometer before sequencing.

Ribosomal RNAs from both eukaryotic and prokaryotic origins were removed from the total RNA samples. The remaining RNA was fragmented to an average size of 250–300 bp and reverse transcribed into double‐stranded cDNA. The cDNA fragments underwent end repair, A‐tailing, and adapter ligation, followed by size selection and PCR amplification. The resulting libraries were subjected to quality control before sequencing. Library preparation was performed using the Novogene NGS RNA Library Prep Set (PT042).

Library quality was evaluated using a Qubit 3.0 fluorometer (Thermo Fisher Scientific, USA) and real‐time PCR for quantification, as well as a Bioanalyzer 2100 (Agilent Technologies, USA) to assess fragment size distribution. Libraries that passed the quality check were pooled according to effective concentration and required data output.

Sequencing was performed on the Illumina NovaSeq X‐Plus platform, which uses Sequencing by Synthesis technology (Guo et al. 2010). In this approach, fluorescently labeled dNTPs, DNA polymerase, and adapter‐specific primers are introduced into a flow cell for clonal amplification. Incorporation of each labeled nucleotide generates a fluorescence signal that is detected by the sequencer and translated by onboard software into sequence reads corresponding to each DNA fragment.

Data analyses were carried out in R (version 4.5.1) using RStudio. Differential gene expression was assessed using the DESeq2 package, and data visualization included principal component analysis (PCA), volcano plots, and heatmaps. Functional enrichment analyses were performed using clusterProfiler, ReactomePA, and fgsea to explore Gene Ontology (GO), KEGG, and Reactome pathway enrichment. Weighted gene co‐expression network analysis (WGCNA) was applied to identify condition‐associated gene modules. Data handling and visualization were performed using ggplot2, pheatmap, dplyr, tibble, tidyr, forcats, and stringr (Yu et al. 2012; Yu and He 2016; Wickham et al. 2019; R Core Team 2023; Korotkevich 2021; Kolde 2022; RStudio Team 2022).

### 2.10 SOS Response Assays

To assess if 5‐FdC induced SOS response, a promoter‐reporter gene construct in *S. aureus* strain USA300 JE2 was used to quantify expression of *recA* (P*recA‐gfp* reporter), as previously described (Clarke et al. 2019; Ha and Edwards 2021). Briefly, we performed three‐fold dilutions of 5‐FdC in a flat‐bottomed, black‐walled 96‐well plate containing MHB that was inoculated with a 1/10 dilution of a stationary‐phase culture of the P*recA‐gfp* reporter strain. The plates were placed into an Infinite M200‐PRO microplate reader (Tecan, Switzerland). Cultures were grown for 15 h at 37°C (700 rpm), with absorbance measured at 600 nm and GFP fluorescence recorded every 15 min. GFP fluorescence was then normalized by OD_600_ data at each time point to account for differences in cell density. To compare SOS response activation, we used a ciprofloxacin control (at 32 μg/mL), a well‐established inducer of SOS response.

We further tested 5‐FdC with a panel of SOS response mutants of *S. aureus* USA300 JE2 supplied by the Network on Antimicrobial Resistance in *S. aureus* (NARSA) program (Table 1). We proceeded to prepare two‐fold dilutions of 5‐FdC in both MHB and Trypticase Soy Broth (TSB; VWR, UK), and wells were finally inoculated with the mutant strains in stationary phase at a final concentration of 1 × 10^5^ CFU/mL. Plates were incubated overnight at 37°C under static conditions. The next day, we compared the MIC obtained with the *S. aureus* USA300 JE2 strain to assess any possible difference.

### 2.11 Statistical Analysis

Statistical analyses and graph plotting were performed using GraphPad Prism (version 8.0.1; GraphPad Software, USA). Normality and homoscedasticity were verified before conducting one‐way or two‐way ANOVA, followed by Dunnett's multiple comparison post hoc test, with statistical significance set at *p* ≤ 0.05.

## 3 Results

### 3.1 High‐Throughput Screening

**Figure 1:**
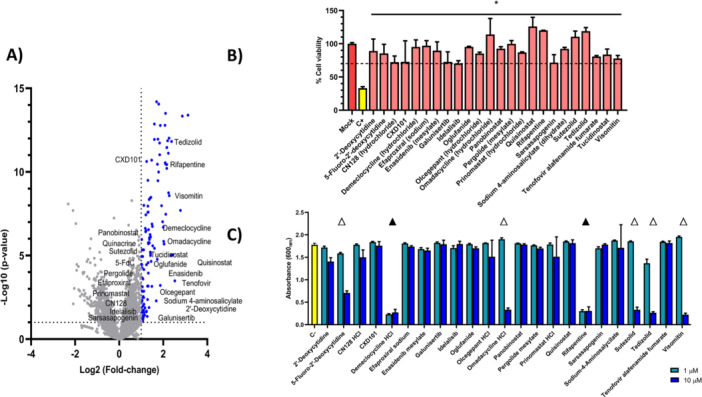
(A) Screening of selected compounds for direct antibacterial activity against *S. aureus* USA300 LAC at 10 µM. Volcano plot shows Log_2_ of the fold‐change in host cell viability relative to infected and untreated controls versus statistical significance (‐Log_10_ *p*‐value). (B) Independent validation of HTS hits. Twenty‐four compounds restored A549 cell viability to > 70% following infection with *S. aureus* USA300 LAC. Gentamicin was included to eliminate extracellular bacteria, ensuring that protection reflected intracellular activity. Statistical significance was determined relative to infected controls, *p*‐value ≤ 0.01*. (C) Known antibiotics (demeclocycline, rifapentine, omadacycline, tedizolid, and sutezolid) showed expected antimicrobial effects, with demeclocycline and rifapentine being the most potent. Visomitin and 5‐FdC, although not developed as antibiotics, displayed significant activity at 10 µM. White triangle: *p*‐value ≤ 0.01 at 10 µM; black triangle: *p*‐value ≤ 0.01 at 1 and 10 µM. Statistical significance was determined relative to an untreated control.

We performed a high‐throughput screen of 6995 compounds at 10 µM, sourced from two partially overlapping drug libraries that included repurposing candidates and natural products (Table S1A). Out of the 6297 unique compounds tested, 317 (5.03%) restored the viability of infected A549 cells to above 70% (corresponding to a log_2_ fold change of 1 relative to the infection control), while 118 (1.87%) achieved viability levels equal to or greater than those of the positive control (Figure 1A; Table S1). We excluded compounds with documented resistant *S. aureus* strains (Table S1B), those unsuitable for oral delivery, and those that restored viability above 140% due to potential oncogenic risk. Following these filters, 28 candidate compounds remained; 24 of these were retained after an *in silico* toxicity assessment via pkCSM and were subsequently revalidated in independent assays with A549 cells (Figure 1B; Table S2) (Pires et al. 2015).

### 3.2 Direct Antimicrobial Activity Assessment

The selected compounds were then screened for direct antibacterial activity against *S. aureus* USA300 LAC (Figure 1C). Known antibiotics in the panel (demeclocycline, rifapentine, omadacycline, tedizolid, and sutezolid) exhibited their expected antimicrobial effects. Among these, demeclocycline and rifapentine showed the highest potency. Notably, visomitin and 5‐FdC, which were not originally developed as antibiotics, demonstrated significant antibacterial activity at 10 µM.

Based on these findings, four compounds, demeclocycline, rifapentine, visomitin, and 5‐FdC, were selected for MIC determination against a panel of *S. aureus* strains: USA300 LAC (CA‐MRSA), USA300 JE2 (plasmid‐cured CA‐MRSA derivative, NTML parental strain), NCTC 13626 (HA‐MRSA), ATCC 25923 (MSSA), and NCTC 8325 (MSSA; Table 1). *S. aureus* USA300 JE2 was the most susceptible strain, while NCTC 13626 exhibited the highest resistance. Demeclocycline was ineffective against NCTC 13626, whereas rifapentine showed consistent activity across all strains tested (Table S3 and Figure S2).

Time‐kill assays with *S. aureus* USA300 JE2 were used to assess bactericidal kinetics. Visomitin eradicated bacteria within 4 h at the highest concentration and within 24 h at a concentration of 2.5 µM. 5‐FdC significantly reduced bacterial counts after 24 h, confirming bactericidal activity. Rifapentine and demeclocycline mainly exhibited bacteriostatic effects under these conditions (Figure S3).

**Figure 2:**
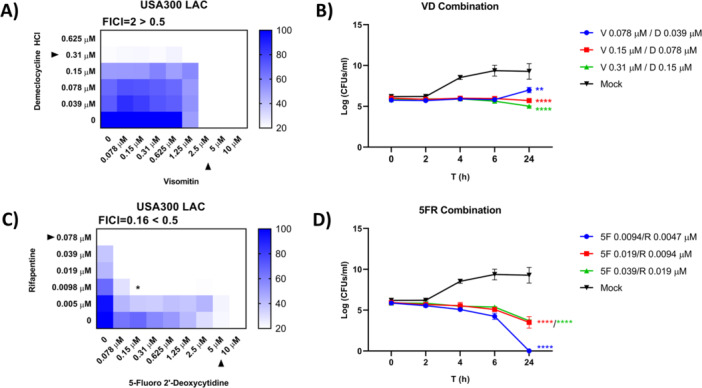
(A, C) Pairwise combination testing against *S. aureus* strains. Asterisks denote synergistic interactions, while black triangles indicate the individual MICs of the compounds tested. (B, D) Antimicrobial killing assays of drug combinations against *S. aureus*. The rifapentine–5‐FdC pair showed enhanced bactericidal activity, eradicating viable bacteria at combinatorial MICs within 24 h. In contrast, the visomitin–demeclocycline combination remained predominantly bacteriostatic. Statistical significance was determined by comparison with mock at 24 h; significance arising from bacterial overgrowth was not considered. *p*‐value ≤ 0.001**, *p*‐value ≤ 0.0001***, *p*‐value ≤ 0.00001****.

Pairwise combination testing against *S. aureus* USA300 JE2 revealed synergy only between rifapentine and 5‐FdC (FICI = 0.33). The visomitin–demeclocycline combination showed additive effects. Further testing across additional *S. aureus* strains demonstrated that rifapentine–5‐FdC synergy persisted in clinical isolates USA300 LAC and NCTC 13626, was additive in NCTC 8325, and indifferent in ATCC 25923 (Table S4 and Figure S4). The visomitin–demeclocycline combination, however, failed to replicate additive effects and exhibited antagonism in some strains (Figure 2A,C, Figures S4 and S5, Table S4).

Antimicrobial killing assays confirmed enhanced bactericidal activity for the rifapentine–5‐FdC combination, eradicating viable bacteria at combinatorial MICs within 24 h. The visomitin–demeclocycline pair remained predominantly bacteriostatic (Figure 2B,D).

### 3.3 Intracellular Infection Efficacy Assessment

To assess the effectiveness of demeclocycline, rifapentine, visomitin, and 5‐FdC as monotherapy without gentamicin, different concentrations were tested. The host cell viability (mCherry) and bacterial viability (GFP fluorescence) were measured to observe how the drugs affected both cellular restoration and bacterial killing (Figure S6). Overall, rifapentine and 5‐FdC were more effective in eradicating intracellular *S. aureus* than demeclocycline or visomitin. Therefore, rifapentine and 5‐FdC were selected for subsequent analyses.

**Figure 3:**
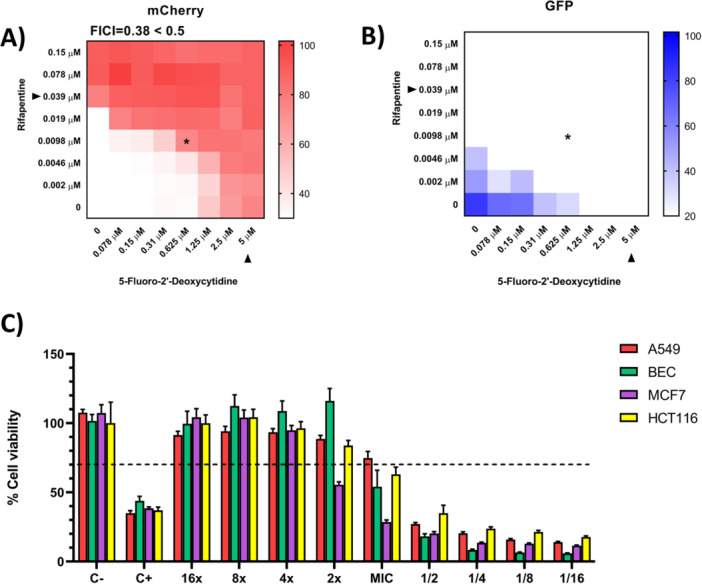
Synergistic activity of rifapentine and 5‐FdC against intracellular *S. aureus*. (A–B) Checkerboard assays in infected A549 cells demonstrated synergy (FICI ≤ 0.5) at 0.625 µM 5‐FdC combined with 0.009 µM rifapentine. Asterisk (*) denotes synergy. (C) Cross‐cell line validation of the rifapentine–5‐FdC combination. HCT116 cells mirrored the synergistic effect observed in A549. Immortalized bronchial epithelial cells (BEC) showed enhanced recovery, with viability restored above 100% at 2× MIC. In contrast, MCF7 cells required 4× MIC to achieve comparable levels of restoration.

Combination testing of rifapentine and 5‐FdC in A549 cells demonstrated intracellular synergy against *S. aureus* (FICI ≤ 0.5) at 0.625 µM 5‐FdC and 0.009 µM rifapentine (Figure 3A,B). To assess whether this effect was consistent across host cell types, parallel assays were performed in MCF7, HCT116, and BEC. HCT116 cells showed a response comparable to A549, whereas BEC cells exhibited restored viability exceeding 100% at two times the MIC. In contrast, MCF7 cells required four times the MIC to achieve similar levels of restoration (Figure 3C).

### 3.4 In Vivo Infection Assays

**Figure 4:**
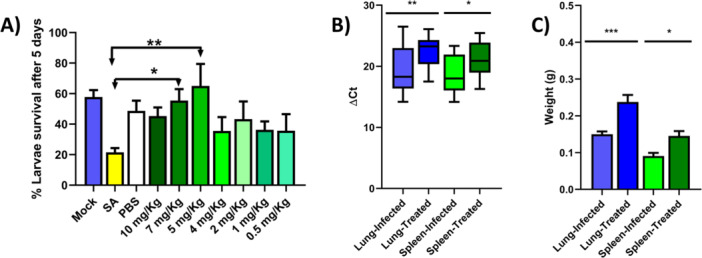
In vivo efficacy of the 5‐FdC plus rifapentine combination. (A) *G. mellonella* infection model. Dose–response testing demonstrated that 5FR protected larvae from *S. aureus* USA300 LAC infection, with doses as low as 5 mg/kg maintaining survival rates comparable to uninfected controls. (B) Murine intranasal infection model; qPCR quantification of *S. aureus* DNA in lungs and spleens revealed significantly lower bacterial burdens in 5FR + THU–treated mice compared with PBS controls. (C) Lung and spleen weights from the same animals, showing increased tissue weights in 5FR + THU–treated groups, consistent with reduced pathogen‐associated damage. Data represent mean ± SEM; *p*‐value ≤ 0.05*, *p*‐value ≤ 0.001**, *p*‐value ≤ 0.0001***. Statistical significance was determined by comparison with respective infected controls.

To evaluate in vivo efficacy, we first tested the synergistic combination of 5‐fluoro‐2′‐deoxycytidine and rifapentine (5FR) using the *G. mellonella* infection model. This system offers several advantages, including incubation at human physiological temperature, low cost, rapid turnaround, and established validation for *S. aureus* infection (Asai et al. 2022; Admella and Torrents 2023). Successful infection was defined as ≥ 80% larval mortality within 5 days post‐infection, providing a robust baseline for assessing antimicrobial efficacy (Figure S7; Hofkens et al. 2024). This level of mortality was consistently achieved using overnight cultures of *S. aureus* USA300 LAC adjusted to a final concentration of 1 × 10^5^ CFU/mL. Dose‐response testing of the 5FR combination revealed that larvae treated with doses as low as 5 mg/kg maintained survival rates comparable to uninfected controls, whereas suboptimal doses conferred only partial protection (Figure 4A).

In a murine intranasal infection model, male and female mice treated with the 5FR combination displayed significantly reduced bacterial burdens in lungs and spleens compared with PBS‐treated controls, as determined by qPCR quantification of *S. aureus* DNA (Figure 4B). In parallel, infected mice receiving 5FR exhibited a significant increase in lung and spleen weights, consistent with reduced pathogen‐associated tissue damage and improved host recovery (Figure 4C). These findings confirm the in vivo efficacy of the 5FR regimen.

### 3.5 Metabolomics Assay

**Figure 5:**
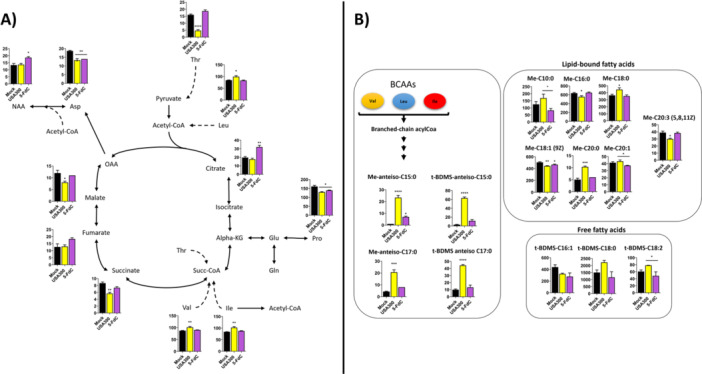
Metabolomic profiling of host cells following *S. aureus* infection and 5‐FdC treatment. (A) Metabolite analysis of A549 cells revealed significant alterations in amino acid and central carbon metabolism upon infection. Infected cells exhibited depletion of threonine, proline, succinate, malate, and aspartate, with threonine levels restored by 5‐FdC treatment to those of uninfected controls. Branched‐chain amino acids (leucine, isoleucine, and valine) were elevated in infected cells but returned to baseline following treatment. Citrate and N‐acetyl‐aspartic acid (NAA) were selectively increased in treated cells, suggesting metabolic reprogramming. (B) Organic phase analysis identified bacterial fatty acid biomarkers. Anteiso‐C15:0 and anteiso‐C17:0, hallmark membrane components of *S. aureus*, were enriched in infected cells but markedly reduced after treatment. Straight‐chain C15:0 and C17:0 species showed no significant differences, consistent with potential host or serum origin. Together, these profiles highlight both partial restoration of host metabolism and a reduction in bacterial signatures following 5‐FdC treatment. *p*‐value ≤ 0.05*, *p*‐value ≤ 0.001**, *p*‐value ≤ 0.0001***, *p*‐value ≤ 0.00001****. Statistical significance was determined by comparing *S. aureus* USA300‐infected cells with mock, and 5‐FdC with both *S. aureus* USA300‐infected cells and mock.

Metabolomic analysis revealed significant alterations in host cell metabolism following *S. aureus* infection in A549 cells, with partial restoration after 5‐FdC treatment. Infected cells exhibited a pronounced depletion of threonine, accompanied by reductions in other amino acids and TCA intermediates, including proline, succinate, malate, and aspartate. Treatment with 5‐FdC restored threonine levels to those observed in uninfected controls, suggesting normalization of amino acid metabolism (Figure 5A). In addition, branched‐chain amino acids (BCAAs) ‐leucine, isoleucine, and valine‐ were elevated in infected cells compared to both treated and uninfected groups, which displayed similar baseline levels. Following treatment, BCAA concentrations returned to levels comparable to uninfected cells (Figure 5B). Citrate levels were significantly elevated in treated cells relative to both infected and uninfected conditions, potentially reflecting enhanced mitochondrial activity or metabolic reprogramming. Additionally, N‐acetyl‐aspartic acid (NAA) was selectively increased in treated cells (Figure 5A). Overall, these findings suggest that 5‐FdC treatment partially restores metabolic homeostasis disrupted by *S. aureus* infection, particularly within amino acid and central carbon metabolism.

Metabolite profiling of the organic phase provided independent evidence for the presence of *S. aureus* in infected host cells (Figure 5B). Among the most significant features were branched‐chain fatty acids typical of *S. aureus*, notably anteiso‐C15:0 and anteiso‐C17:0, which are known to constitute major components of the bacterial membrane and play a role in regulating membrane fluidity under changing conditions (Sen et al. 2016; Frank et al. 2021). Both anteiso fatty acids were strongly enriched in infected cells, whereas their levels were markedly reduced upon treatment, consistent with a decrease in the intracellular bacterial load (Figure 5B). In contrast, straight‐chain C15:0 and C17:0 species did not show significant changes, suggesting that they may be derived from host metabolism or serum components. The detection of these bacterial fatty acid markers thus provides robust, independent validation of the antimicrobial efficacy observed in our in vitro and in vivo infection models.

### 3.6 RNA‐Sequencing

**Figure 6:**
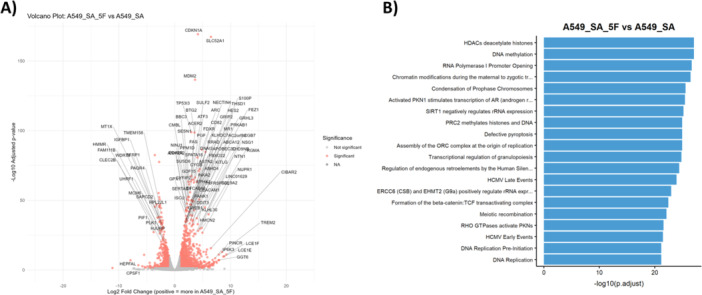
Transcriptomic response of A549 host cells to *S. aureus* infection and 5‐FdC treatment. (A) Volcano plot showing the distribution of differentially expressed host genes (log_2_ fold change vs. –log_10_ *p*‐value). Significantly upregulated and downregulated genes are highlighted in red. (B) Reactome pathway enrichment analysis of differentially expressed genes. Enriched pathways included many related to DNA damage response, consistent with 5‐FdC–induced replication stress.

Global transcriptional profiling of infected A549 cells treated with 5‐FdC revealed substantial changes in gene expression across multiple functional categories (Figure 6; Table S5). Uniform Manifold Approximation and Projection (UMAP) analysis demonstrated a clear separation between infected and untreated or 5‐FdC‐treated cells (Figure S8) (McInnes et al. 2018). This separation suggests that 5‐FdC‐induced effects are distinct from baseline *S. aureus* infection responses, reflecting targeted pharmacological modulation of host cellular pathways.

Genes involved in lipid metabolism and cholesterol transport (e.g., *ABCG4*) were upregulated, consistent with membrane remodeling processes known to influence bacterial entry and intracellular survival (Safi et al. 2023). At the same time, strong activation of metabolic stress pathways was evident through the upregulation of *CHAC1*, *DDIT3 (CHOP)*, *ATF4*, and *NUPR1*, indicative of an active integrated stress response (ISR), enhanced amino acid metabolism, oxidative stress signaling, and the unfolded protein response (UPR; Table S5) (Grootjans et al. 2016; Wortel et al. 2017; Sun et al. 2024). Stress‐responsive transcription factors such as *ATF3* were also induced, reflecting a broad defensive shift toward inflammatory and stress‐adaptation programs (Du et al. 2022).

Key regulators of proliferation, including *ATAD2* and *AGTR1*, were downregulated, and GO analyses confirmed a coordinated repression of genes involved in mitotic progression, DNA replication initiation, and nucleotide biosynthesis (Figure S10). These changes indicate that 5‐FdC redirects biosynthetic resources away from proliferation and toward cellular defense processes (Assmann and Finlay 2016).

Reactome and GO pathway analyses highlighted robust enrichment of DNA‐damage response (DDR) and stress‐adaptation pathways (Figures S9 and 6B). A large set of execution‐phase DNA‐repair genes, including *CHEK2*, *TP53*, *APEX2*, *OGG1*, *POLB*, *ERCC1/4*, *XRCC1*, *LIG4*, *SIRT6*, and *GADD45G*, was upregulated (Figure S11), consistent with active engagement of repair mechanisms following DNA damage (Deplanche et al. 2019; Berkova et al. 2025). Given that *S. aureus* is a genotoxic bacterium that triggers double‐strand breaks through phenol‐soluble modulins (Berkova et al. 2025), this enhanced repair signature suggests that 5‐FdC amplifies host repair responses beyond those typically seen during infection.

In contrast, checkpoint sensors and transducers, including *ATM*, *ATR*, *CHEK1*, *BRCA1/2*, *RAD51/54*, *TP53BP1*, *CDKN1A*, and *WEE1*, were markedly downregulated. This indicates a shift away from ATM/ATR‐dependent checkpoint arrest toward checkpoint‐independent p53 activation and rapid repair execution (Smith et al. 2010; Patil et al. 2013; Berkova et al. 2025). Downregulation of *BLM*, *MRE11*, *UNG*, and *LIG3* further supports a transition from slow, cell‐cycle‐dependent homologous recombination toward faster repair mechanisms such as BER and SSB repair (Mjelle et al. 2015; Hindi et al. 2021).

Concomitantly, pro‐apoptotic genes (*CASP3*, *CASP9*, *BAX*, *PMAIP1*) were upregulated, suggesting that 5‐FdC primes infected cells for controlled apoptosis. This constitutes a host‐protective mechanism, as apoptosis eliminates the intracellular niche available to *S. aureus* (Behar and Briken 2019; Mulcahy et al. 2020; Rodríguez‐González and Gutiérrez‐Kobeh 2024). Interestingly, despite apoptotic priming, 5‐FdC restores cell viability, indicating a finely balanced “repair‐or‐die” program in which cells prioritize damage resolution but retain a conditional apoptotic failsafe.

5‐FdC treatment also induced a strong type I interferon signature, including *IFNA1, IFNB1, STAT1, STAT2, OAS1, ISG15*, and *IFIT*‐family genes (Figure 6B) (Snyder et al. 2017; Lee and Ashkar 2018; Mazewski et al. 2020). These pathways are commonly activated by cytosolic DNA generated during DNA‐damage repair via cGAS‐STING or TLR signaling (Amadio et al. 2021; Song et al. 2022). The downregulation of negative regulators such as *SOCS3, PRKDC, IRF3, IRF7, BST2*, and anti‐apoptotic *BCL2* further suggests that 5‐FdC relieves immunosuppressive constraints, amplifying both interferon‐driven antimicrobial signaling and apoptotic clearance (Mahony et al. 2016; Lan et al. 2020).

### 3.7 SOS Response

**Figure 7:**
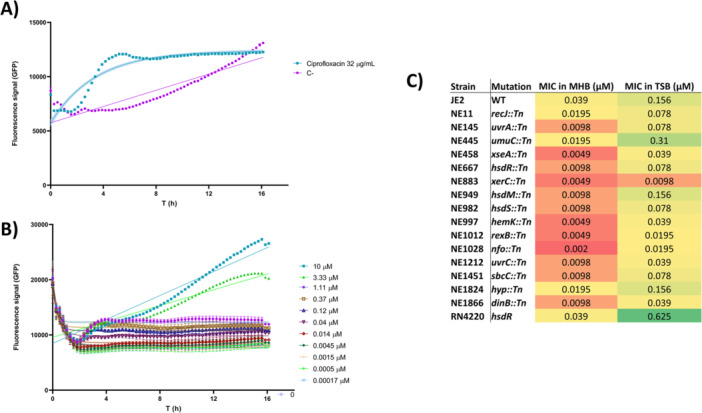
DNA damage induction by 5‐FdC in *S. aureus*. (A, B) SOS response monitoring in *S. aureus* USA300 JE2 harboring a *PrecA‐gfp* reporter, with ciprofloxacin as a positive control. 5‐FdC induced a dose‐dependent increase in GFP fluorescence, surpassing ciprofloxacin at certain concentrations. Untreated bacteria displayed a gradual fluorescence increase attributed to stationary‐phase starvation stress. (C) Minimum inhibitory concentrations of 5‐FdC for *S. aureus* USA300 JE2 and DNA repair–deficient transposon mutants were determined in MHB (low thymidine) and TSB (high thymidine).

We assessed the ability of 5‐FdC to induce DNA damage in *S. aureus*. Using a *S. aureus* USA300 JE2 wild‐type strain harbouring a *PrecA‐gfp* reporter, we monitored SOS response induction, with ciprofloxacin as a positive control. 5‐FdC induced a dose‐dependent SOS response, surpassing ciprofloxacin at certain doses. An increase in fluorescence in untreated bacteria over time was attributed to DNA damage that typically occurs as cells enter the stationary phase (Clarke et al. 2019; Ha and Edwards 2021) (Figure 7A,B).

Further, MICs were determined for a panel of *S. aureus* USA300 JE2 transposon mutants defective in DNA repair, in both MHB and TSB. MHB's low thymidine content potentiates thymidine‐less death, whereas TSB rescues bacteria with exogenous thymidine, allowing dissection of DNA damage mechanisms (Ha et al. 2020). As expected, wild‐type MICs were higher in TSB compared to MHB, reflecting thymidine rescue (Figure 7C). Mutants in recombinational repair (*rexB*, NE1012; *recJ*, NE11), nucleotide excision repair (*uvrA*, NE145; *uvrC*, NE1212), and end‐processing enzymes (*xerC*, NE883; *nfo*, NE1028; *sbcC*, NE1451; *xseA*, NE458) exhibited strong hypersensitivity, particularly in MHB. Mutants in translesion synthesis (*dinB*, NE1866; *umuC*, NE445) also showed increased susceptibility, supporting a role for SOS‐mediated lesion bypass. In addition, strains defective in restriction‐modification systems (*hsdR*, NE667; *hsdM*, NE949; *hsdS*, NE982) were more sensitive in MHB, consistent with reduced ability to protect DNA integrity under thymidine‐limiting conditions. The RN4220 laboratory strain, which carries a natural *hsdR* mutation, exhibited similar susceptibility trends but grew more robustly in MHB or TSB, consistent with its adaptation to laboratory conditions and higher metabolic robustness in rich media. Collectively, these data indicate that 5‐FdC exerts its antimicrobial activity by inducing DNA damage that requires multiple repair pathways for bacterial survival.

## 4 Discussion

Drug repurposing is a cost‐effective strategy to accelerate antimicrobial discovery, but most efforts have focused on extracellular pathogens. Intracellular infections remain largely neglected, despite the clinical importance of organisms such as *S. aureus*, which persist in diverse host cell types and evade antibiotic therapy (Lehar et al. 2015). Only a few studies have investigated repurposed drugs in intracellular *S. aureus*. Metabolic profiling–guided selection, functional phenotypic screening, and host‐directed modulation strategies have identified candidate compounds with intracellular activity (Bravo‐Santano et al. 2018; Bravo‐Santano, Capilla‐Lasheras, et al. 2019; Bravo‐Santano, Ellis, et al. 2019; Bravo‐Santano, Stölting, et al. 2019), extending earlier pioneering attempts (Czyż et al. 2014). More recently, targeted host‐directed screens have been applied (Zheng 2025), though the scale of these efforts remains modest compared with viral or parasitic infection studies (Khan et al. 2024; Elfawal et al. 2025).

In this study, we report the largest high‐throughput screen performed to date against intracellular *S. aureus*, testing 6297 compounds. Beyond demonstrating the technical feasibility of large‐scale infection‐based assays in mammalian cells, this work delivers a valuable resource of host‐directed candidates with both direct and combinatorial antibacterial potential.

The 5FR combination showed consistent synergy across distinct *S. aureus* lineages, including community‐acquired MRSA (USA300 LAC) and hospital‐acquired MRSA (NCTC 13626), and its efficacy extended beyond carcinoma‐derived cell lines to a non‐tumorigenic bronchial epithelial model, providing a more physiologically relevant context for airway colonization. These results supported the progression from in vitro studies to animal infection models.

At the same time, our data indicate that the potency of 5FR is not uniform across all *S. aureus* strains or host cell types. While synergy is maintained in MRSA strains, the combination displays only additive or indifferent effects in MSSA isolates, and different host cell lines show distinct dose‐response thresholds. This variability is consistent with the ability of intracellular *S. aureus* to rewire its metabolism and stress responses according to the intracellular niche, and with the fact that host cell types differ in basal metabolic state, nutrient availability, and activation of cytoprotective pathways (Bravo‐Santano et al. 2018). One possible explanation for this lineage‐specific synergy is the distinct physiological burden associated with methicillin resistance. MRSA strains carry SCCmec elements that impose metabolic and regulatory costs, reshaping basal stress‐response and metabolic programs and increasing dependence on DNA repair and adaptive stress pathways (Collins et al. 2010). These constraints may render MRSA more vulnerable to the combined perturbations imposed by 5‐FdC and rifapentine, whereas MSSA strains, lacking these resistance‐associated burdens, maintain greater physiological robustness and therefore exhibit predominantly additive or indifferent responses. Although further work is required to define the underlying mechanism, these observations support the idea that resistance‐associated physiology can modulate intracellular drug interactions. Thus, although 5FR is broadly active, its ultimate efficacy reflects the interplay between pathogen lineage‐specific physiology and the metabolic and stress‐response landscape of the host cell. This context‐dependence underscores the need to evaluate intracellular therapies across diverse host‐pathogen backgrounds (Bravo‐Santano et al. 2018).

Nevertheless, the in vivo efficacy of 5FR was demonstrated first in the *G. mellonella* infection model, a validated invertebrate system that reduces vertebrate use while providing outcomes that correlate with mammalian infection (Tsai et al. 2016; Kavanagh and Sheehan 2018; Sheehan et al. 2019; Piatek et al. 2021). Guided by these results, we advanced to a murine pneumonia model, where the 5FR regimen significantly reduced bacterial burdens in lungs and spleen, was well tolerated, and limited systemic dissemination.

Metabolomic profiling provided additional mechanistic insight into how 5‐FdC modulates the intracellular niche. A consistent signature was the depletion of threonine in infected cells, which we had previously observed in HeLa cells (Bravo‐Santano et al. 2018) and linked to AMPK activation and induction of autophagy. Restoration of threonine levels by 5‐FdC suggests that the compound may alleviate starvation‐driven autophagy, thereby enhancing antibacterial responses. Other changes, including altered branched‐chain amino acids and bacterial fatty acid markers, independently confirmed the reduction of intracellular *S. aureus* (Kaiser et al. 2016; Sen et al. 2016; Mahmud et al. 2020; Frank et al. 2021). RNA‐seq further showed that 5‐FdC activates host stress responses. The strong hypersensitivity of DNA repair mutants, together with SOS induction in a *recA* reporter, indicates that 5‐FdC exerts direct antimicrobial activity through multifaceted DNA damage beyond thymineless death (Clarke et al. 2019; Oe et al. 2020; Clarke et al. 2021; Ha and Edwards 2021; Ledger et al. 2023; Cheng et al. 2024). Together, these findings support a dual mechanism in which 5‐FdC directly damages bacterial DNA while also reshaping host metabolic and stress pathways to restrict the intracellular environment.

Recent evidence indicates that host DNA‐damage stress responses can influence the outcome of *S. aureus* infection. Under baseline infection conditions, *S. aureus* activates the ATM–CHK2–p53 axis, which can trigger apoptosis in severely damaged cells; this apoptotic route effectively eliminates the intracellular niche required for bacterial survival (Berkova et al. 2025). Beyond apoptosis, DDR activation also induces broader transcriptional and metabolic reprogramming, which can reduce cellular permissiveness. Yet, the contributions of cell‐cycle arrest or senescence to antibacterial defense are context‐dependent and less well defined.

In our study, however, 5‐FdC did not reinforce canonical ATM/CHK2 signaling. Instead, it suppressed *ATM, ATR* and *CHEK1* transcription while preserving or enhancing *TP53* expression, indicating a shift toward checkpoint‐independent p53 activation coupled to accelerated repair and conditional apoptotic clearance. This mode of DDR rewiring aligns with the broader concept that enhancing host stress‐responsive pathways can drive infected cells into a non‐permissive state. For example, activation of the mitochondrial unfolded protein response via the SATB2/DVE‐1 axis enhances resistance to *S. aureus* and other intracellular pathogens by reshaping metabolic flux and limiting nutrient availability (Cui et al. 2025).

Together, our results support a comprehensive model wherein 5‐FdC treatment reprograms *S. aureus*‐infected cells from a checkpoint‐arrested, permissive state toward an active “repair‐or‐clear” response characterized by: i) enhanced DNA repair through sustained upregulation of execution‐phase repair genes; ii) interferon‐mediated immunity via robust type I IFN and JAK‐STAT pathway activation; iii) controlled apoptosis through pro‐apoptotic gene induction and removal of anti‐apoptotic constraints; iv) metabolic redirection toward amino acid biosynthesis and oxidative stress management. This coordinated activation of DDR, immune signaling, and apoptotic machinery advances the emerging concept that pharmacological modulation of the DDR, particularly through agents that enhance DNA sensing and immune priming while bypassing checkpoint‐mediated arrest, can favor host‐directed clearance of intracellular bacterial infections (Berkova et al. 2025).

In this context, rifapentine, which is unsuitable as monotherapy due to rapid resistance (Sterling et al. 2011), gains markedly enhanced efficacy. When combined with 5‐FdC, rifapentine acts on bacteria already metabolically stressed and engaged in DNA repair, resulting in robust synergy across clinical isolates and multiple host cell types, overcoming a common limitation of repurposing studies that rely on laboratory strains (Shapira et al. 2024). Such synergy reduces effective doses, limits off‐target toxicity, and lowers resistance risk (Dillon et al. 2019). Importantly, host‐directed therapies expand therapeutic targets beyond bacterial pathways (Kaufmann et al. 2018), and our findings demonstrate that combining them with conventional antibiotics can restrict intracellular *S. aureus* on two fronts. Although synergistic antibiotic–antibiotic, antibiotic–antibiofilm, and host–pathogen pairings have been reported (Farha et al. 2015; Minato et al. 2018; Shi et al. 2022; Zheng et al. 2022; Deusenbery et al. 2023; Folliero et al. 2023; Sharma and Gutheil 2023), their activity does not always extend to intracellular contexts, where drug penetration and host physiology are major determinants (Buyck et al. 2013). In our study, however, the 5‐FdC‐rifapentine combination retained intracellular synergy in multiple cell types, including non‐tumorigenic epithelial cells, supporting its translational relevance. The persistence of intracellular reservoirs contributes to immune evasion, chronic and relapsing infections, and treatment failure (Day et al. 2024; Lathram and Radka 2025). Yet only ~1% of repurposing studies have addressed intracellular pathogens (Lorente‐Torres et al. 2024).

## 5 Conclusions

This study reports the largest repurposing screen performed to date against intracellular *S. aureus*, demonstrating the feasibility and value of large‐scale infection‐based drug discovery. From 6297 compounds, we identified a synergistic combination between the nucleoside analogue 5‐fluoro‐2′‐deoxycytidine and rifapentine that acts through coordinated host–pathogen targeting. This combination was active across multiple *S. aureus* lineages, including MRSA, diverse mammalian cell types, and two in vivo infection models, supporting its broad applicability.

Mechanistic analyses revealed that 5‐FdC simultaneously induces bacterial DNA damage and reprograms host metabolic and stress‐response pathways, thereby creating a cellular environment that restricts intracellular bacterial survival and enhances rifapentine efficacy. These dual effects illustrate how manipulating host responses can potentiate conventional antimicrobials against pathogens that persist within protected intracellular niches.

Together, our findings provide a strong proof‐of‐concept for rational combination therapies that integrate host‐directed and pathogen‐directed mechanisms. They highlight host–pathogen dual targeting as a promising strategy to overcome the therapeutic limitations of current antibiotics and pave the way for new interventions against multidrug‐resistant intracellular pathogens.

## Author Contributions

**Blanca Lorente‐Torres:** investigation, writing – original draft, formal analysis, data curation, validation, visualization, writing – review and editing. **Helena Á. Ferrero:** investigation, writing – review and editing, methodology, validation, visualization, formal analysis, data curation. **Pablo Castañera:** investigation, methodology, validation, writing – review and editing, formal analysis. **Jesús Llano‐Verdeja:** investigation, visualization, formal analysis, writing – review and editing. **Sergio Fernández‐Martínez:** investigation, visualization, writing – review and editing, formal analysis. **Amanda Herrero‐González:** investigation, visualization, writing – review and editing, formal analysis, methodology, supervision, data curation. **Farzaneh Javadimarand:** investigation, visualization, writing – review and editing, formal analysis. **Roberto López:** visualization, writing – review and editing, formal analysis, project administration, supervision, resources, data curation. **Jesús F. Aparicio:** supervision, resources, project administration, writing – review and editing, funding acquisition. **Andrew M. Edwards:** resources, supervision, writing – review and editing, funding acquisition. **Volker Behrends:** resources, supervision, data curation, formal analysis, software, validation, writing – review and editing, methodology, investigation. **Luis M. Mateos:** investigation, funding acquisition, writing – review and editing, visualization, supervision, resources, project administration. **Álvaro Mourenza:** investigation, writing – review and editing, visualization, validation, methodology, software, formal analysis, data curation, supervision, project administration. **Michal Letek:** conceptualization, investigation, funding acquisition, writing – review and editing, visualization, validation, methodology, software, formal analysis, project administration, resources, supervision, data curation.

## Ethics Statement

Ethical approval for this study was obtained from the *Universidad de León* and the *Junta de Castilla y León* (ETICA‐ULE‐060‐2021, ETICA‐ULE‐003‐2023, ETICA‐ULE‐043‐2023, and OEBA‐ULE‐001‐2025) for work involving biosafety level 2 pathogens, genetically modified human cell lines, and the *Galleria mellonella* and mouse infection models.

## Conflicts of Interest

The authors declare no conflicts of interest.

## Declaration of Generative AI and AI‐Assisted Technologies in the Writing Process

During the preparation of this work, the authors used Grammarly and Perplexity to improve language and readability. After using these tools, the authors reviewed and edited the content as needed and take full responsibility for the publication's content.

## Supporting information

Supporting File 1

Supporting File 2

Supporting File 3

Supporting File 4

Supporting File 5

Supporting File 6

## Data Availability

The data that support the findings of this study are available in the supplementary material of this article.
