# Supplementary material for: Host–Pathogen Dual Targeting With Repurposed Drugs Identifies a Synergistic Therapy for Intracellular Staphylococcus aureus
Source: Microbiologyopen. 2026 May 28;15(3):e70317. doi: 10.1002/mbo3.70317 (PMC13239213; doi:10.1002/mbo3.70317)
Supplement: Supplementary file 3 — Supporting File 3 [file MBO3-15-e70317-s003.docx]

**Table S3.** MICs of 5-FdC, demeclocycline HCl, rifapentine, and visomitin against a panel of *S. aureus* strains.

| **Strains** | **5-FdC** | **Demeclocycline HCl** | **Rifapentine** | **Visomitin** |
| --- | --- | --- | --- | --- |
| *ATCC 25923* | >10 µM | 0.625 µM | 0.039 µM | 2.5 µM |
| *NCTC 8325* | 10 µM | 0.31 µM | 0.0195 µM | 1.25 µM |
| *USA300 JE2* | 0.039 µM | 0.15 µM | 0.0195 µM | 2.5 µM |
| *USA300 LAC* | 5 µM | 0.31 µM | 0.078 µM | 2.5 µM |
| *NCTC 13626* | 10 µM | >10 µM | 0.0195 µM | 5 µM |
