# Supplementary material for: Host–Pathogen Dual Targeting With Repurposed Drugs Identifies a Synergistic Therapy for Intracellular Staphylococcus aureus
Source: Microbiologyopen. 2026 May 28;15(3):e70317. doi: 10.1002/mbo3.70317 (PMC13239213; doi:10.1002/mbo3.70317)
Supplement: Supplementary file 4 — Supporting File 4 [file MBO3-15-e70317-s005.docx]

**Table S4.** Pairwise drug combination testing against *S. aureus* strains. Synergy between rifapentine and 5-FdC (FICI = 0.33) was observed in *S. aureus* USA300 JE2 and persisted in USA300 LAC and NCTC 13626. Additive, indifferent, or antagonistic effects were detected depending on the strain and drug pair.

|  | **5FR** | |  |
| --- | --- | --- | --- |
| **Strains** | **5-FdC** | **Rifapentine** | **FICI** |
| *ATCC 25923* | 2.5 µM | 0.0195 µM | 1.250 |
| *NCTC 8325* | 1.25 µM | 0.00975 µM | 0.625 |
| *USA300 JE2* | 0.00975 µM | 0.0024 µM | 0.330 |
| *USA300 LAC* | 0.078 µM | 0.00975 µM | 0.266 |
| *NCTC 13626* | 0.625 µM | 0.0048 µM | 0.309 |
|  | **VD** | |  |
| **Strains** | **Visomitin** | **Demeclocycline HCl** | **FICI** |
| *ATCC 25923* | 2.5 µM | 0.31 µM | 1.496 |
| *NCTC 8325* | 0.31 µM | 0.31 µM | 2.129 |
| *USA300 JE2* | 0.15 µM | 0.039 µM | 0.560 |
| *USA300 LAC* | 2.5 µM | 0.31 µM | 2.567 |
| *NCTC 13626* | 2.5 µM | >10 µM | 2.000 |
