# Supplementary material for: Host–Pathogen Dual Targeting With Repurposed Drugs Identifies a Synergistic Therapy for Intracellular Staphylococcus aureus
Source: Microbiologyopen. 2026 May 28;15(3):e70317. doi: 10.1002/mbo3.70317 (PMC13239213; doi:10.1002/mbo3.70317)
Supplement: Supplementary file 6 — Supporting File 6 [file MBO3-15-e70317-s004.pdf]

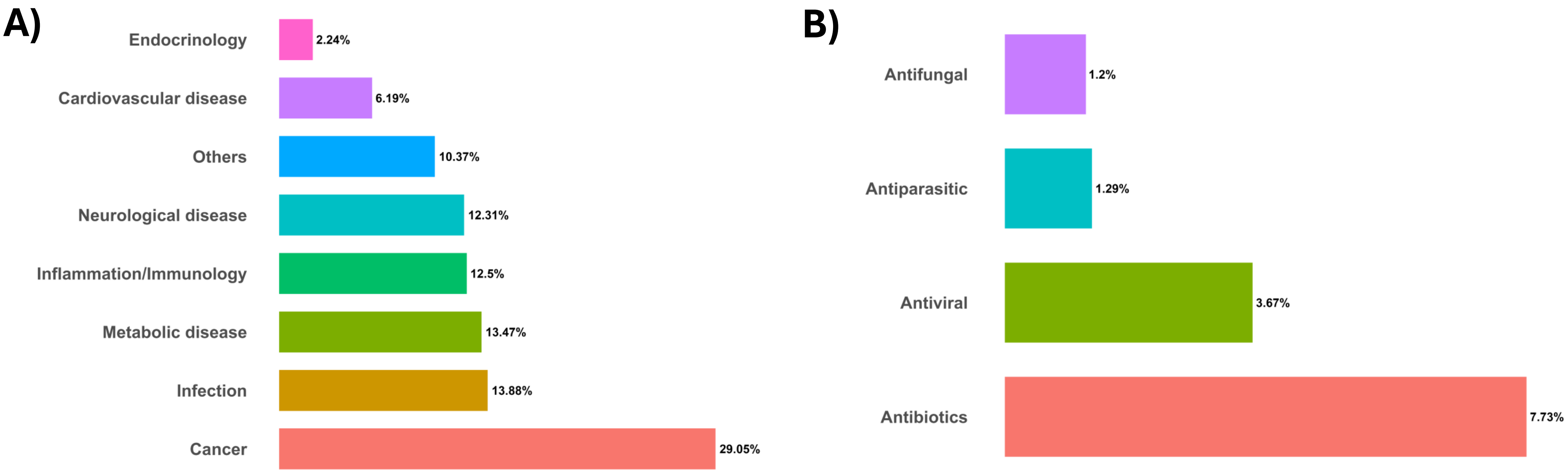

Figure S1. Target analysis of the drug library. (A) Distribution of drugs across major therapeutic areas. (B) Proportion of infection-related targets, including bacterial, viral, parasitic, and fungal mechanisms.

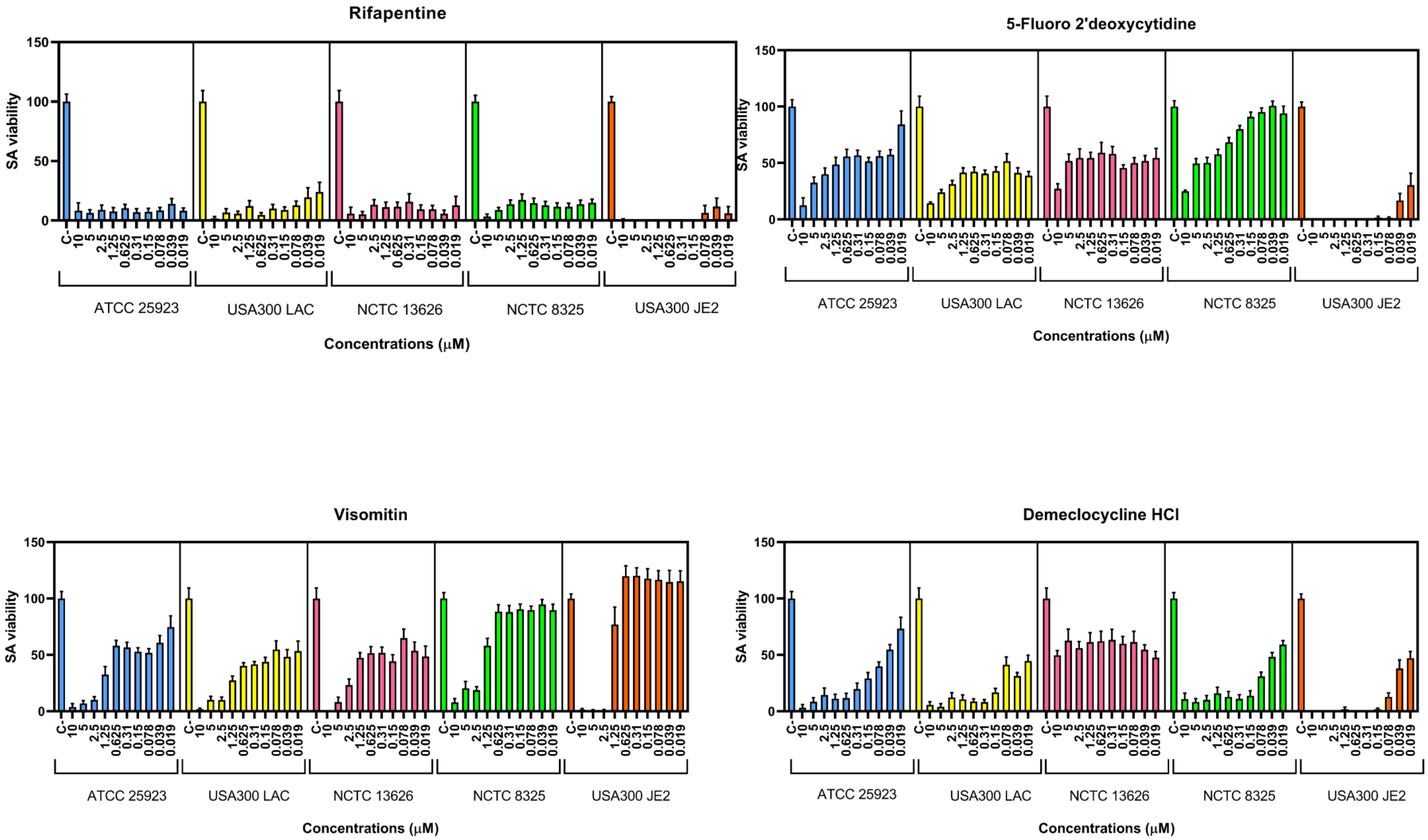

Figure S2. Minimum inhibitory concentrations (MICs) of Demeclocycline HCl, Rifapentine, Visomitin, and 5-FdC against a panel of *S. aureus* strains. Demeclocycline was ineffective against NCTC 13626, whereas Rifapentine exhibited consistent activity across all strains tested.

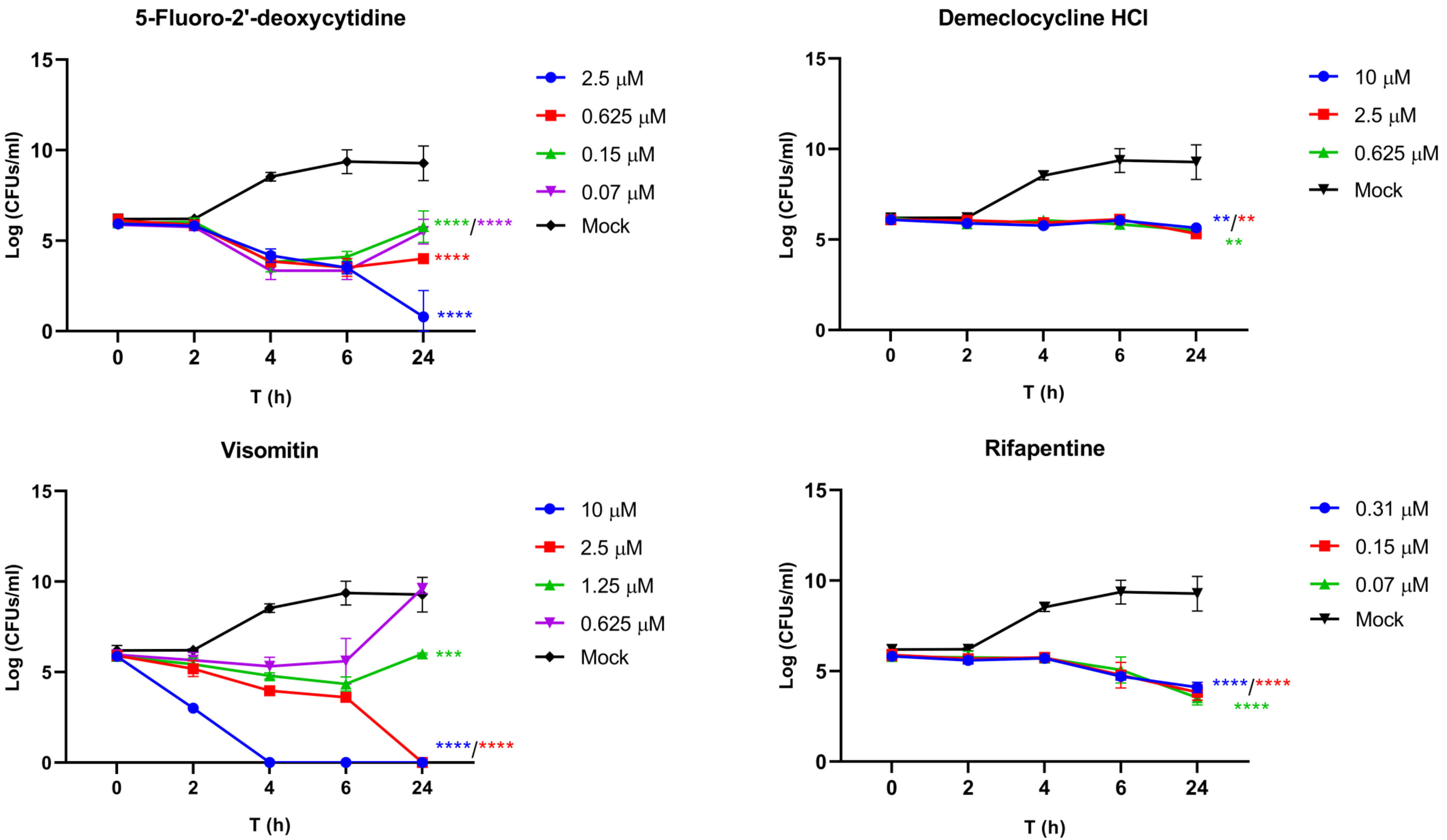

Figure S3. Time-kill assays with *S. aureus* USA300 JE2. Visomitin completely eradicated bacteria within 4 h at the highest concentration and within 24 h at 2.5 µM. 5-FdC significantly reduced bacterial counts after 24 h, confirming bactericidal activity. Rifapentine and Demeclocycline primarily exhibited bacteriostatic effects under these conditions. Statistical significance was determined by comparison with mock at 24 h; p-value ≤ 0.001**; p-value ≤ 0.0001***; p-value ≤ 0.00001****. Significance arising from bacterial overgrowth was not considered.

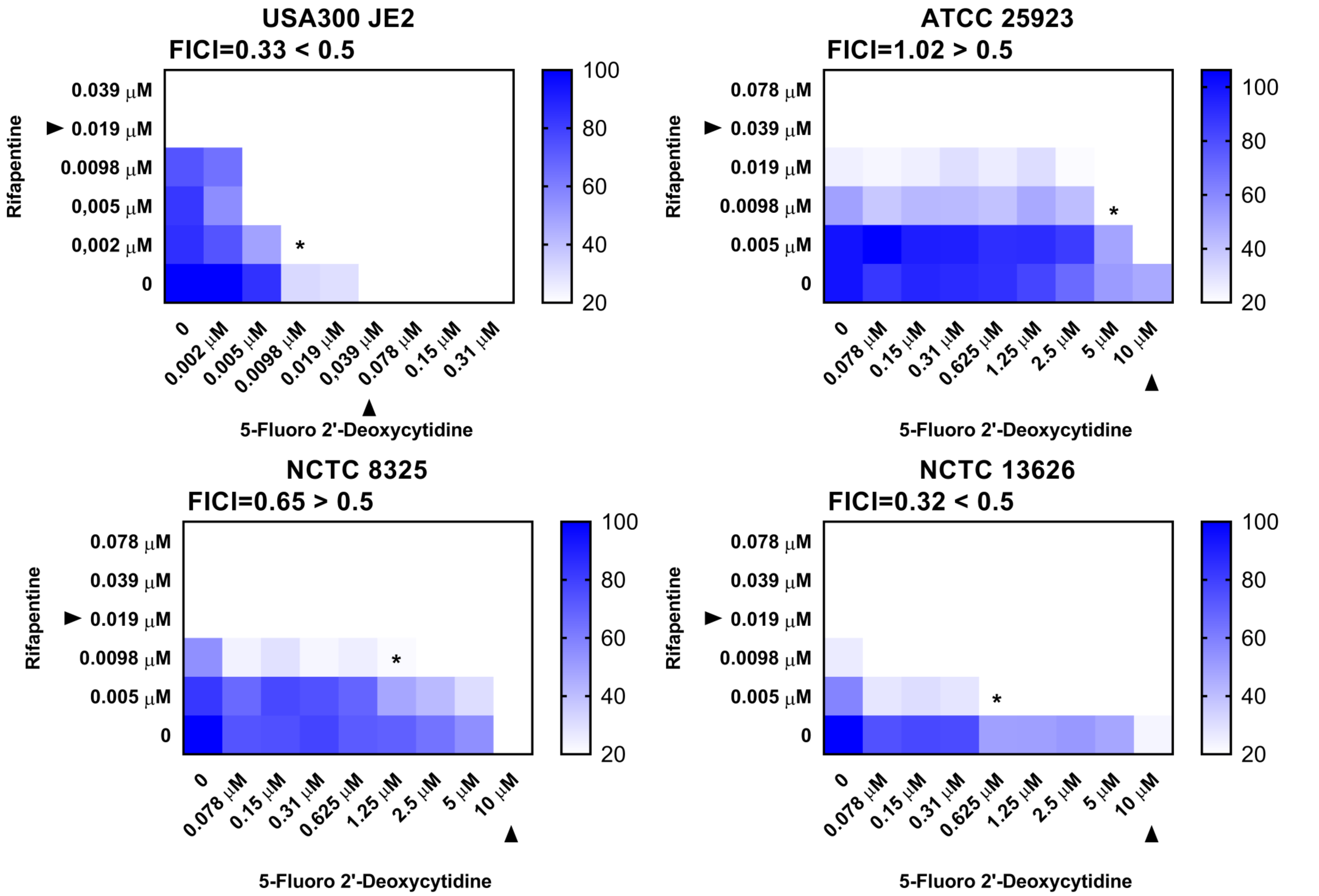

Figure S4. Pairwise combination testing against *S. aureus* strains. Rifapentine–5-FdC displayed synergy in *S. aureus* USA300 JE2 and maintained activity in NCTC 13626. Asterisks denote synergistic or additive interactions, while black triangles indicate the individual MICs of the compounds tested.

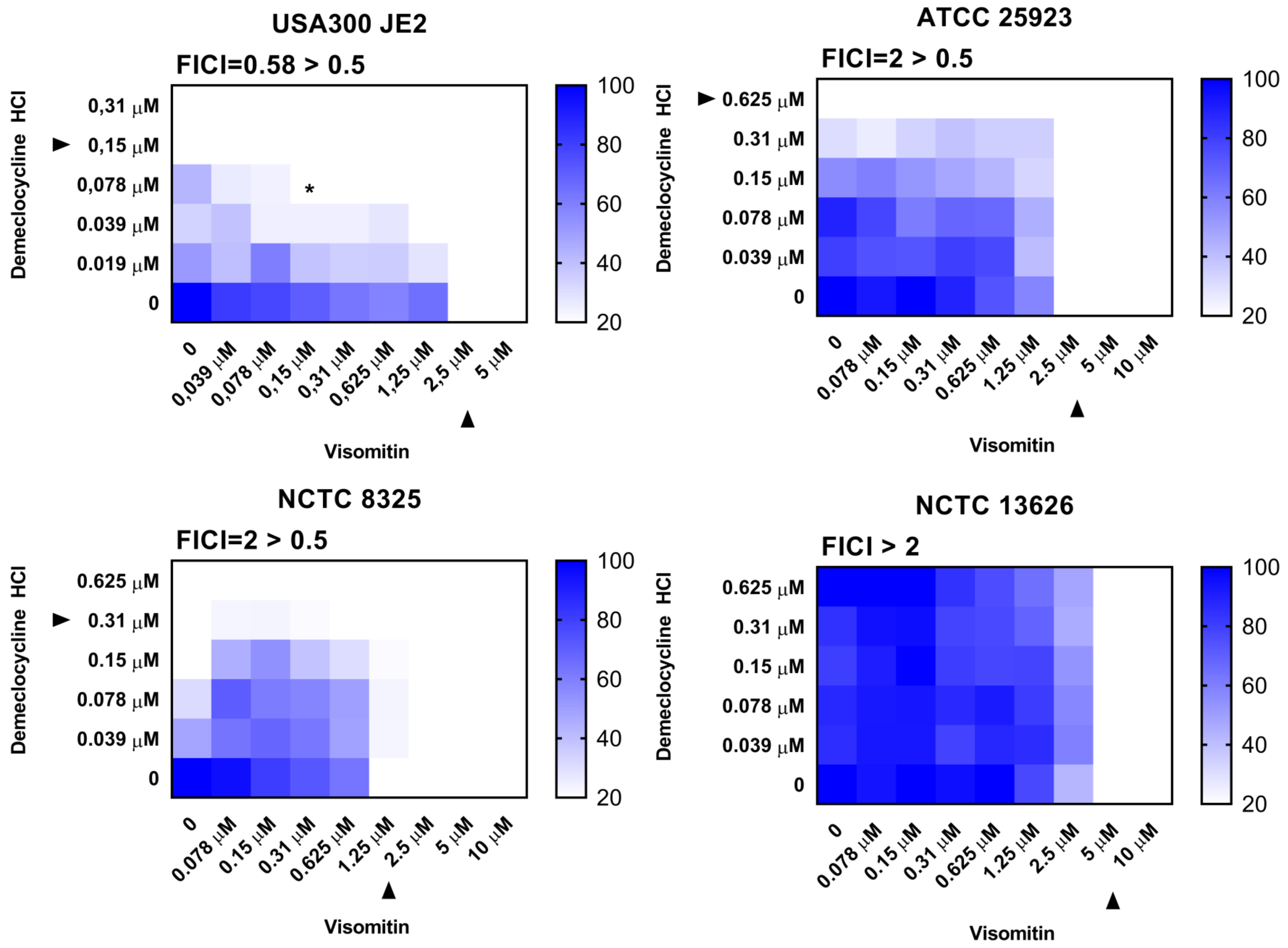

Figure S5. The Visomitin–Demeclocycline combination, although additive in *S. aureus* USA300 JE2, failed to reproduce this effect in other strains and showed antagonism in NCTC 13626. Asterisks denote synergistic or additive interactions, while black triangles indicate the individual MICs of the compounds tested.

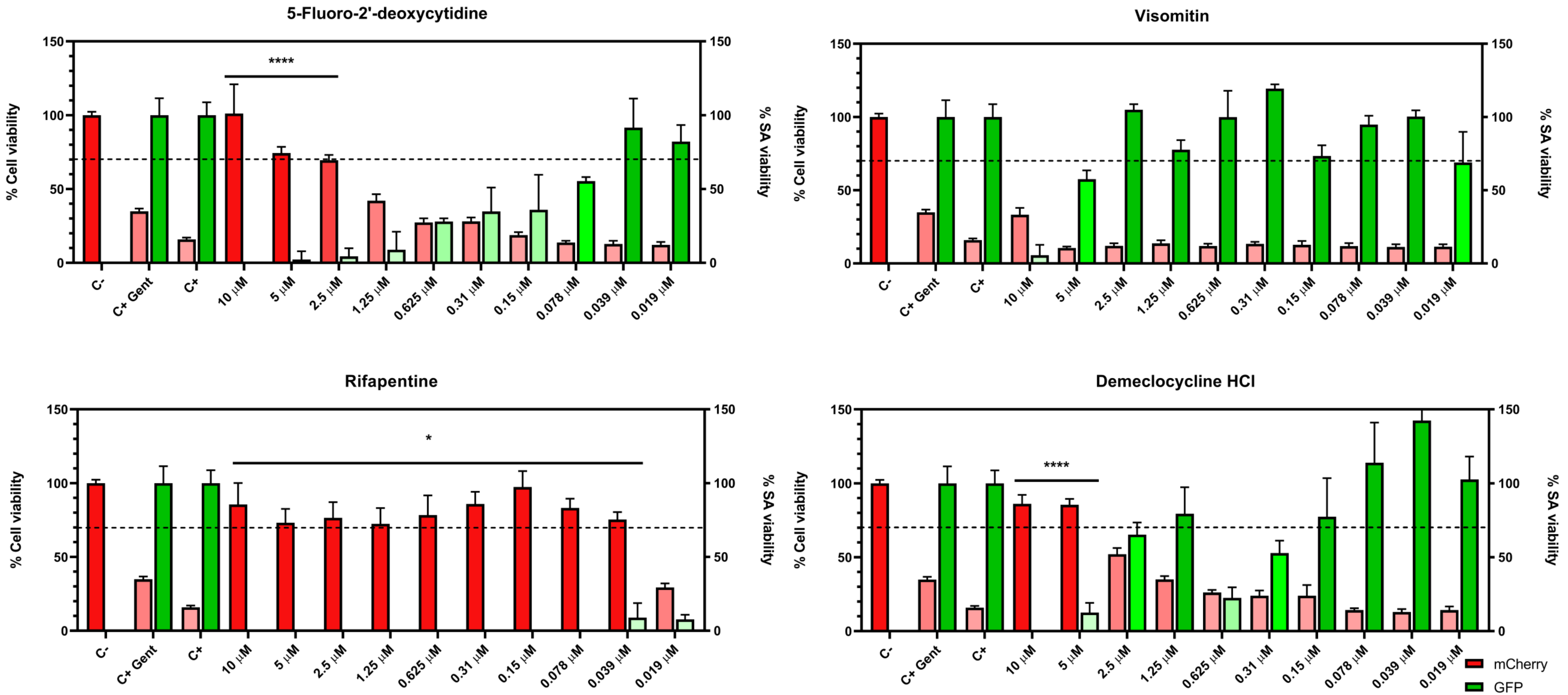

Figure S6. Monotherapy evaluation of candidate compounds against intracellular *S. aureus*. A549 cells infected with GFP-expressing *S. aureus* were treated first with gentamicin to kill extracellular bacteria, and then with increasing concentrations of Demeclocycline HCl, Rifapentine, Visomitin, or 5-fluoro-2'-deoxycytidine. Host cell viability was monitored by mCherry fluorescence, while bacterial viability was quantified by GFP signal. Dose-dependent effects highlight the differential ability of each compound to promote host cell restoration and/or bacterial killing. p-value ≤ 0.05*; p-value ≤ 0.001**; p-value ≤ 0.0001***; p-value ≤ 0.00001****, calculated relative to *S. aureus*-infected controls.

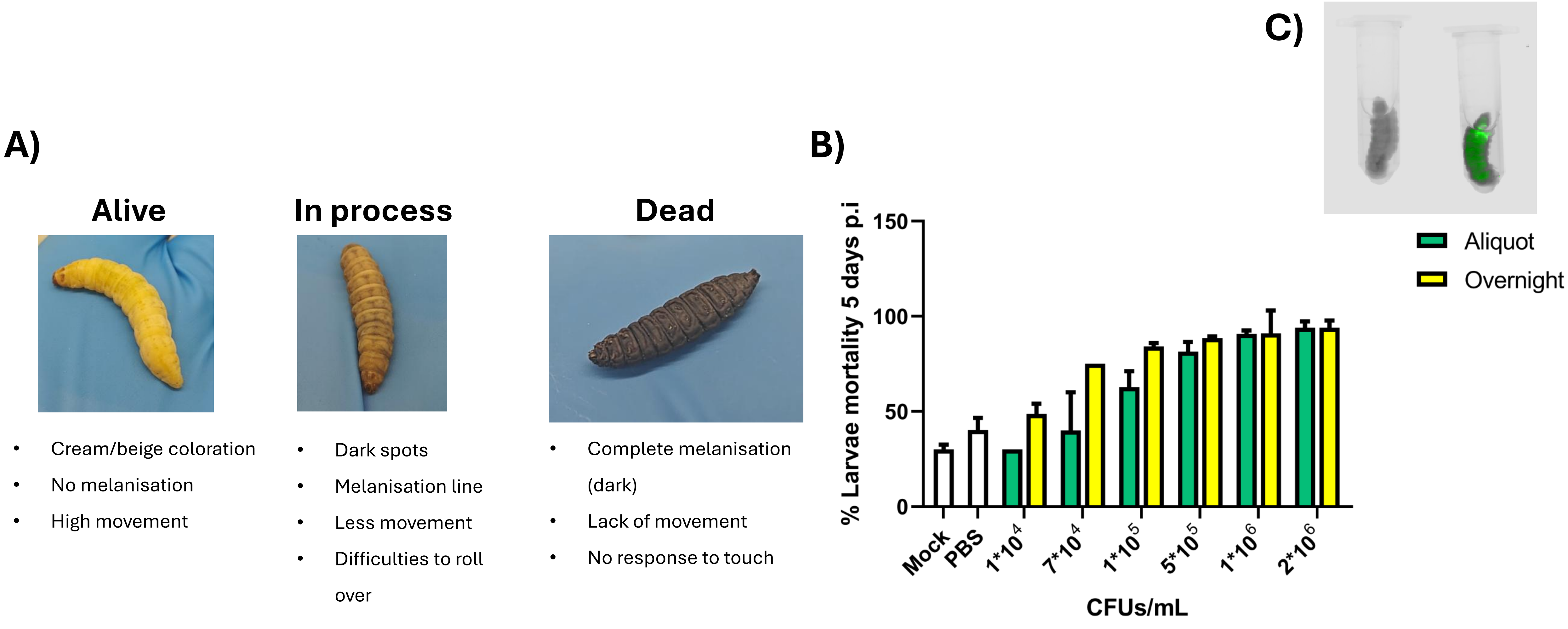

Figure S7. *G. mellonella* infection model with *S. aureus* USA300 LAC strain. (A) Criteria used to assess larval survival versus death. (B) Percentage mortality on day 5 following infection with different concentrations of *S. aureus* USA300 LAC, comparing bacteria from frozen aliquots versus overnight cultures. Controls included unmanipulated larvae (mock) and larvae injected with PBS. Data are presented as mean ± SEM. (C) Fluorescence imaging of larvae using the Odyssey XF® system. Left: PBS-injected control. Right: larvae infected with 10^4 CFU/mL GFP-expressing *S. aureus* USA300. No fluorescence was detected in PBS-injected controls, whereas a strong GFP signal confirmed bacterial colonization in infected larvae.

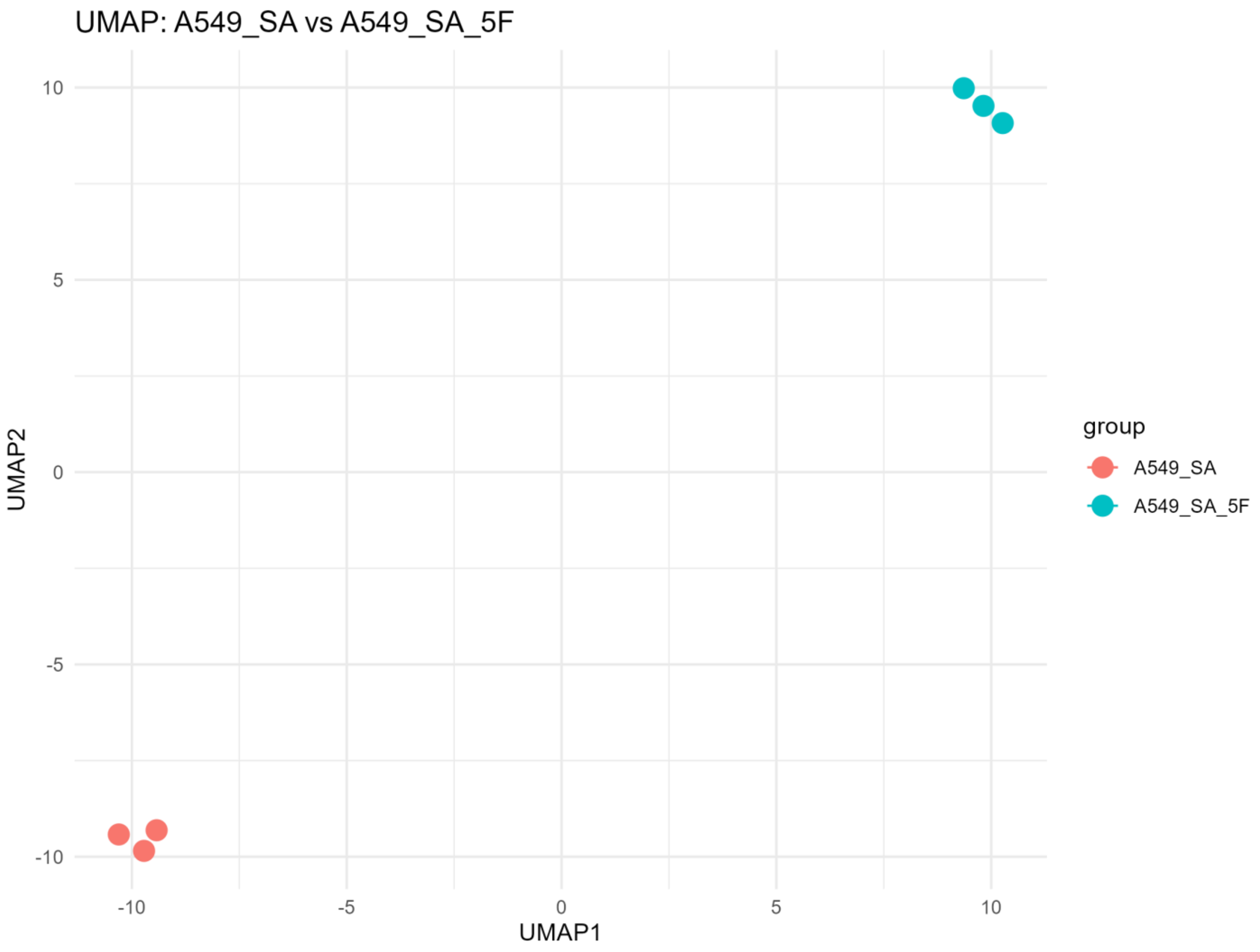

Figure S8. UMAP visualization of transcriptomic profiles from A549 cells infected with *S. aureus* USA300 LAC and treated with 5-fluoro-2'-deoxycytidine. Distinct clustering was observed between infected (red) and treated (blue) samples, indicating marked differences in global gene expression. Replicates within each condition clustered closely together, reflecting consistency of the transcriptional response.

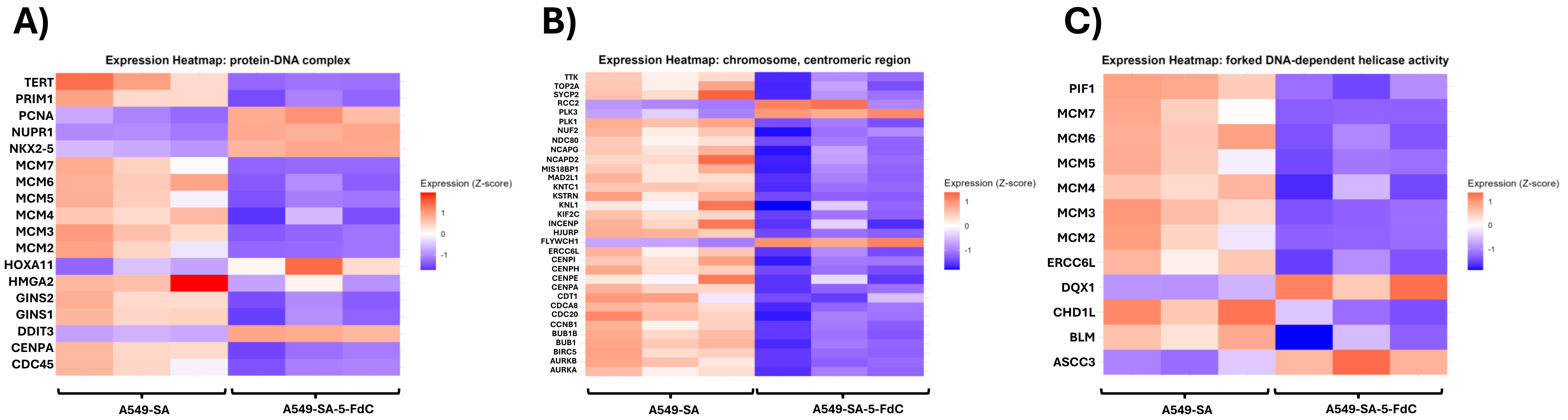

Figure S9. Heatmap representation of transcriptomic changes in A549 cells infected with *S. aureus* USA300 LAC and treated with 5-fluoro-2'-deoxycytidine. Genes associated with (A) protein–DNA complex formation, (B) chromosome centromeric region, and (C) forked DNA-dependent helicase activity were significantly downregulated following treatment. These patterns highlight the inhibitory effect of 5-FdC on DNA replication and mitotic progression, consistent with its role as a nucleoside analogue that induces replication stress.

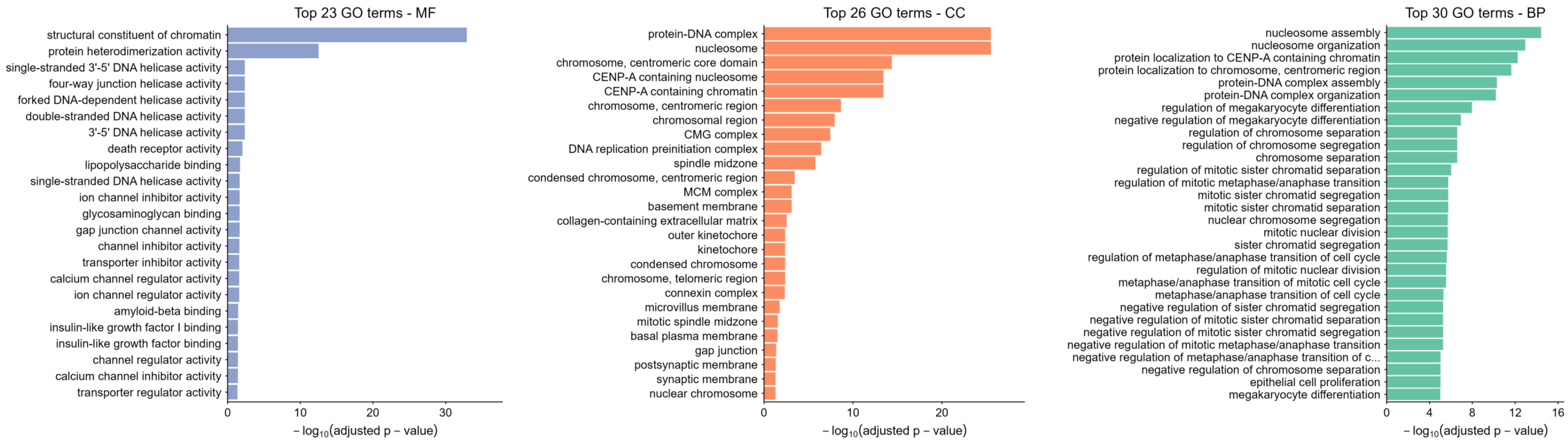

Figure S10. Gene Ontology (GO) enrichment analysis of host transcriptomic changes in A549 cells infected with *S. aureus* and treated with 5-fluoro-2'-deoxycytidine (5-FdC). Molecular Function (MF), Cellular Component (CC) and Biological Process (BP) terms enriched in treated cells, including DNA damage response, nuclear and chromatin-associated complexes, cell cycle arrest, and stress response pathways. Collectively, the data indicate that 5-FdC treatment controls stress response and nucleotide biosynthesis, reshaping host defense and survival programs during *S. aureus* infection.

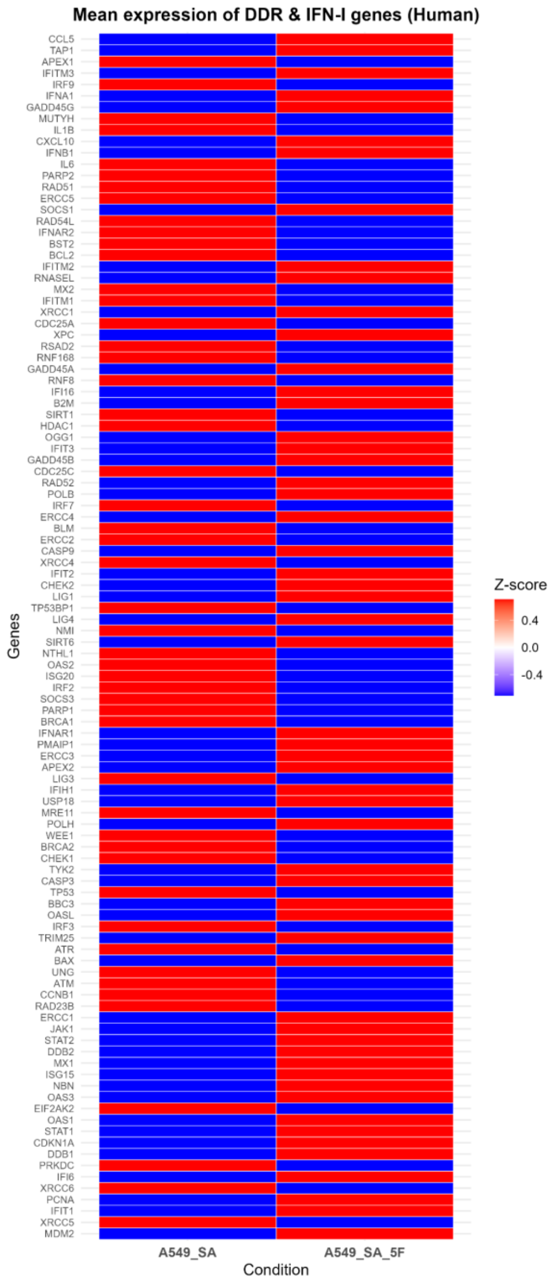

Figure S11. Heatmap showing the mean normalized (Z-score) expression of DNA damage response (DDR) and type I interferon (IFN-I) genes in *S. aureus*-infected untreated A549 cells (A549_SA) or treated with 5-FdC (A549_SA_5F). The figure illustrates broad transcriptional activation of DDR and IFN-I pathways following 5-FdC treatment, consistent with enhanced repair, immune signaling, and apoptotic responses.
